# Identification of predictors based on drug targets highlights accurate treatment of goserelin in breast and prostate cancer

**DOI:** 10.1186/s13578-020-00517-w

**Published:** 2021-01-06

**Authors:** Yue Zhao, Huimin Sun, Jianzhong Zheng, Chen Shao, Dongwei Zhang

**Affiliations:** 1 Department of Urology, Xiang’an Hospital of Xiamen University, School of Medicine, Xiamen University, Xiamen, China; 2 Clinical Central Research Core, Xiang’an Hospital of Xiamen University, School of Medicine, Xiamen University, Xiamen, China; 3 Department of Surgery, The Second Affiliated Hospital of Harbin Medical University, Harbin, China

**Keywords:** Breast cancer, Prostate cancer, Goserelin, Network pharmacology, Prognosis

## Abstract

Goserelin is an effective alternative to surgery or estrogen therapy in prostate cancer palliation, and possibly to ovariectomy in premenopausal breast cancer. However, not all users of goserelin can benefit from it, or some patients are not sensitive to goserelin. The advent of network pharmacology has highlighted the need for accurate treatment and predictive biomarkers. In this study, we successfully to identify 76 potential targets related to the compound of goserelin through network pharmacology approach. We also identified 18 DEGs in breast cancer tissues and 5 DEGs in cells, and 6 DEGs in prostate cancer tissues and 9 DEGs in cells. CRABP2 is the common DEG both in breast and prostate cancer. The risk prediction models constructed with potential prognostic targets of goserelin can successfully predict the prognosis in breast and prostate cancer, especially for very young breast cancer patients. Moreover, seven subgroups in breast cancer and six subgroups in prostate cancer were respectively identified based on consensus clustering using potential prognostic targets of goserelin that significantly influenced survival. The expression of representative genes including CORO1A and ANXA5 in breast and DPP4 in prostate showed strong correlations with clinic-pathological factors. Taken together, the novel signature can facilitate identification of new biomarkers which sensitive to goserelin, increase the using accuracy of goserelin and clarify the classification of disease molecular subtypes in breast and prostate cancer.

## Introduction

Goserelin is a synthetic analogue of gonadotrophin-releasing hormone (GnRH) which stimulates gonadotrophin and sex hormone release in the short term, and then causes suppression with continued administration [1, 2]. Breast and prostate cancer are the most commonly diagnosed nonskin cancer in women and men, respectively [3]. Monthly goserelin depot therapy produces partial disease remission or stabilisation in about 60%-80% of men with previously untreated prostate cancer, a rate equivalent to that achieved with orchidectomy or diethylstilbestrol (stilboestrol) [4]. About 30% to 45% of premenopausal women with breast cancer responded to goserelin using objective assessment criteria, suggesting comparability to ovariectomy [5]. Thus, goserelin is an effective alternative to surgery or estrogen therapy in prostate cancer palliation, and possibly to ovariectomy in premenopausal breast cancer.

Recently, network pharmacology (NP) was proposed as a promising approach to discover drugs from a systems perspective and at the molecular level. It combines bioavailability prediction, multiple drug target prediction and network analysis to understand the active compounds and therapeutic targets of drug [6, 7]. Lee et al. predicted multi-compounds and multi-targets linked to hyperlipidemia and atherosclerosis in Yijin-Tang by Network analysis [8]. These previous studies suggested that NP will be a good predictive tool for exploring the therapeutic targets of goserelin and its relationships with breast and prostate cancer.

Currently, clinicians mainly use the clinic-pathological factors and clinical stages to assess the risk of breast and prostate cancer in patients and guide clinical treatment [9, 10]. These clinic-pathological variables have demonstrated specificity or sensitivity in predicting the prognosis only in some of breast and prostate cancer patients, which gene is the target of goserelin and whether its expression is related to the prognosis is still unknown [11]. In addition, because of the response rates are limited and some of breast and prostate cancer patients do not benefit from goserelin. Therefore, it is essential to develop a new risk assessment system that can effectively predict the prognosis of patients and relate to goserelin sensitivity. In this study, we successfully constructed a risk prediction model with several prognostic genes which linked to compound of goserelin. Through stratifying the risk of patients and their gene expression characteristics, we could find more effectively therapeutic targets associated with goserelin, and preventing poor prognosis in high risk patients.

## Materials and methods

### Data selection and processing

The RNA-Seq gene expression profiles (FPKM format) of patients with breast and prostate cancer were downloaded from the cancer genome atlas (TCGA) portal (https://cancergenome.nih.gov/). It contains 1164 breast samples (1053 breast tumor samples and 111 normal samples) and 551 prostate samples (499 prostate tumor samples and 52 normal samples). The clinical data of above samples, such as gender, age, tumor grade, clinical stage, and survival time, were also downloaded from the TCGA database. The RNA expression data of cells with breast and prostate cancer were downloaded from the Gene Expression Omnibus (GEO, http://www.ncbi.nlm.nih.gov/geo/). Data from the GSE 62410, GSE 107209 datasets were used for differential expression analysis. GSE 62410 [GPL16686 platform, Affymetrix Human Gene 2.0 ST Array] contains 6 samples, including 3 PrEC cells and 3 LNcaP cells. GSE 107209 [GPL17586 platform, Affymetrix Human Transcriptome Array 2.0] contains 6 samples, including 3 MCF-10A cells and 3 UFH-001 cells. The gene expression data and clinical data of very young breast cancer patients were derived from international cancer genome consortium (ICGC) portal (https://dcc.icgc.org/). Genotype-Tissue Expression (GTEx) program (https://commonfund.nih.gov/gtex) established a data resource and tissue bank of healthy people. Data of GTEx were used to plot the expression of genes in multiple normal tissues in our study, and we used the R language “ggpubr” package to drawing boxplot to distinguish the expression of genes between male and female. PharmMapper Server is designed to identify potential target candidates for the given probe small molecules (drugs, natural products, or other newly discovered compounds with binding targets unidentified) using pharmacophore mapping approach [12]. The 2D or 3D structure of goserelin candidate compound was collected from pubchem database (https://pubchem.ncbi.nlm.nih.gov/), and finding the best interaction mode between the potential target candidates and compound by pharmacophore database (http://www.lilab-ecust.cn/pharmmapper/). R software (3.6.2) was used for data extraction and sorting to obtain the gene expression matrices and clinical data.

### Differential expression analysis

To identify differentially expressed genes (DEGs) between tumor and normal tissues/cells, we used the R language “limma” package to screen the DEGs. Mann Whitney test was performed to determine differential expression levels of genes between tumor samples and corresponding control samples. |log2 fold change (FC)|> 1, and false discovery rate (FDR) values < 0.05 were considered to be statistically significant.

### Construction of a drug regulatory network and functional enrichment analysis

A protein protein interaction (PPI) network of related goserelin target genes were constructed using the STRING online database (https://stringdb.org/) [13], and the confided score with correlation degree > 0.400 was as the cut-off value to obtain a network. Compound-compound target network construction was performed by the network visualization software Cytoscape (http://www.cytoscape.org/) [14]. Gene ontology (GO) and Kyoto Encyclopedia of Genes and Genomes (KEGG) pathway enrichment analysis were conducted with R language “clusterProfiler” and “enrichplot” package, FDR < 0.05 was set as the threshold. Bubble chart was used to visualize the biological process (BP), cellular component (CC), and molecular function (MF) of GO enrichment. The circle-plot was used to visualize the pathways of KEGG.

### Construction of risk prediction model

In order to build a prognostic model applicable to breast and prostate cancer patients and relate to target genes of goserelin, all target genes were used to conduct a univariate Cox survival regression, and P value < 0.05 was used to create a lasso regression model. This method was applied to reduce potential over-fit and implemented through the “glmnet” R package. Univariate and multivariate Cox analysis were conducted with R language “forest” and “survival” package. The risk scores of each patient were calculated through the prediction formula of the risk prediction model. We calculated the cut-off value used to determine whether the patient is at high or low risk. The formula used for this model was:

$${\text{Risk score}} = \sum\limits_{i = 1}^{{\text{n}}} {gene_{i} } \cdot coef_{i}$$

### Verification of the validity of the risk prediction model

Recurrence-free survival (RFS) was collected and defined as the interval from the data of surgery to the end of follow-up results or death. Overall survival (OS) was calculated from the date of diagnosis to the date of death or last follow-up. Survival curves were estimated using the Kaplan–Meier method, and the log-rank test was used to test for differences between groups. The time-dependent receiver operating characteristic (tdROC) curve analysis was first applied to evaluate the predictive accuracy of the model for cancer-specific death or biochemical recurrence based on the risk scores, with the help of the “survivalROC” R package. Chi-squared test or Fisher’s exact test were used to investigate the correlation between risk model and clinicopathologic variables, and draw the heatmap through the “pheatmap” R package, *P* < 0.05 was considered statistically significant for all tests.

### Identification of molecular subtypes using consensus clustering

Consensus clustering was performed using the “ConsensusClusterPlus” package in R to identify subgroups based on the target genes of goserelin. This algorithm determined consensus clustering by measuring the stability of clustering results from the application of a given clustering method to random subsets of data. In each iteration, 80% of the tumors were sampled, and the k-means algorithm with the Euclidean squared distance metric was used. These results were compiled over 100 iterations. After executing ConsensusClusterPlus, we obtained the cluster consensus and item-consensus results. Graphical output results included heatmaps of the consensus matrices, which displayed the clustering results, consensus cumulative distribution function (CDF) plots, and delta area plots, and which allowed us to determine an approximate number of clusters. Numbers of clusters were determined according to the following criteria: relatively high consistency within the cluster, relatively low coefficient of variation, and no appreciable increase in the area under the CDF curve. Associations between both clinicopathologic characteristics and clustering were analyzed using the χ^2^ test or Fisher’s exact test, and draw the heatmap through the “pheatmap” R package, *P* < 0.05 was considered statistically significant for all tests.

### Analysis of the representative target gene

We are screening the representative target genes through the Venn diagram (http://bioinformatics.psb.ugent.be/webtools/Venn/). We inputted the retained candidate target genes into the UALCAN (http://tumorsurvival.org/index.html) database. Wilcoxon signed-rank test was used to generate a *P*-value for expression of age, gender, stages, different molecular subtypes, nodal metastasis status, menopause status, or gleason sorces analysis, all *P*-values < 0.05 were considered statistically significant.

## Results

### Goserelin regulatory networks construction and enrichment analysis

**Fig. 1:**
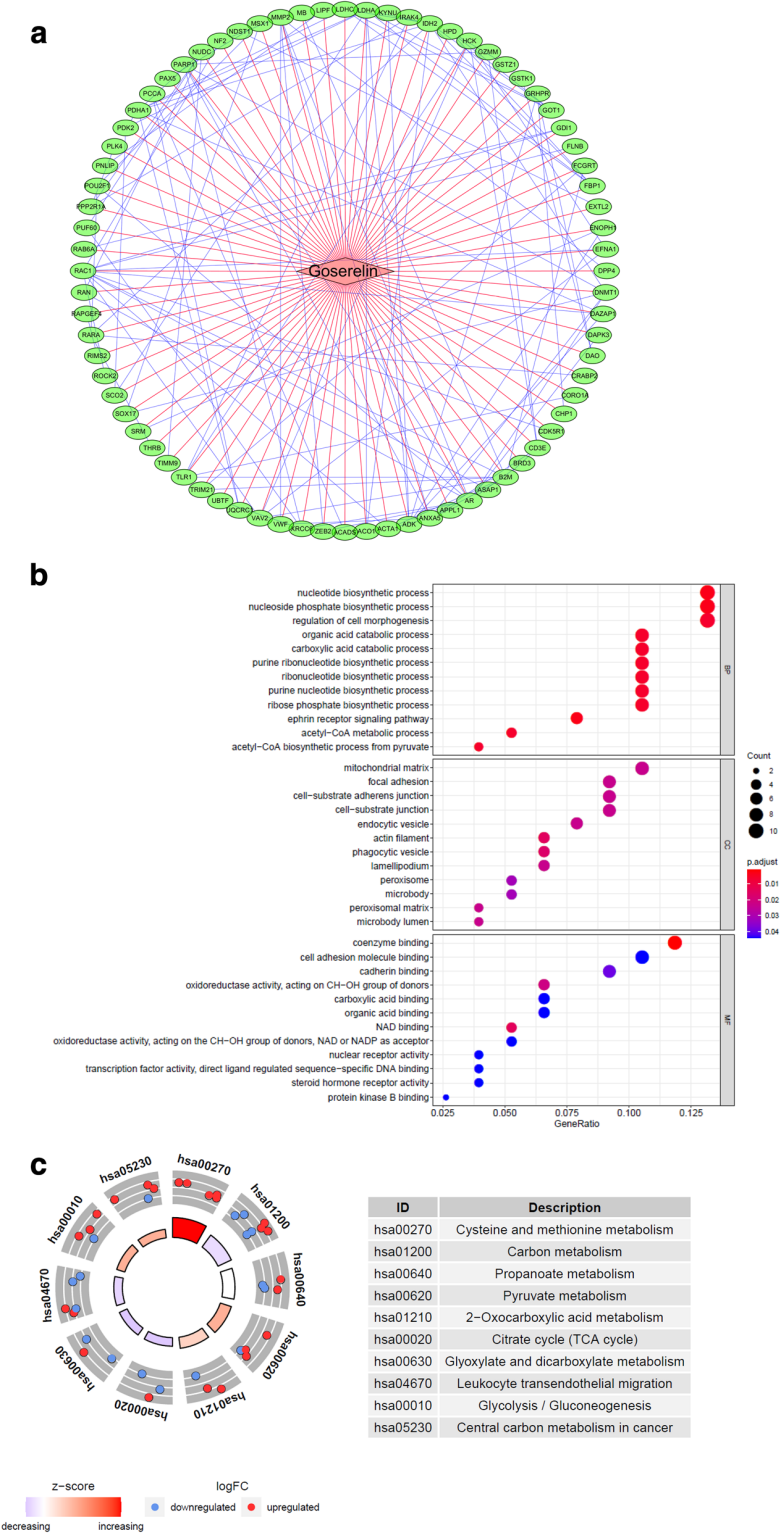
Regulatory networks construction and enrichment analysis of goserelin target genes. **a** Network of the interactions among the 76 targets predicted for goserelin. Red lines show compound and target genes interactions, and blue lines show interactions shared by target genes. **b** Bubble-plot of GO enrichment in cellular component terms, biological process terms and molecular function terms. **c** Circle-plot of KEGG enriched terms

Chemical structures of goserelin was download in pubchem portal (Additional file 1: Figure S1a). To identify synergistic and mechanistically related targets for the compound of goserelin, we employed a guilt-by-association analysis on a multilayered molecular interaction network. There are 77 nodes (76 compound target nodes and 1 compound node) and 269 edges composed regulatory network (Fig. 1a). In this network, the relationship between compound and target genes were shown in red lines, and the PPI relationship of the target genes were shown in blue lines. In PPI network, some nodes (AR, MMP2, ANXA5, B2M, CD44, RAC1, IDH2, LDHA, ADK and SHMT2) have higher degrees (Additional file 1: Figure S1b and c).

To explore the biological functions of 76 target genes, they were categorized into BP, CC, and MF. Under stringent threshold conditions (*P*-adjust < 0.05), we identified 202 specific BP, 15 CC, and 21 MF of GO terms were enriched in these genes (Fig. 1b). The outcome of GO enrichment of the genes were shown in Additional file 1: Table S1. Additionally, analysis using clusterProfiler indicated that these genes were significantly enriched in 11 pathways as shown in Fig. 1c (Additional file 1: Table S2).

### Identification of DEGs in breast and prostate cancer

**Fig. 2:**
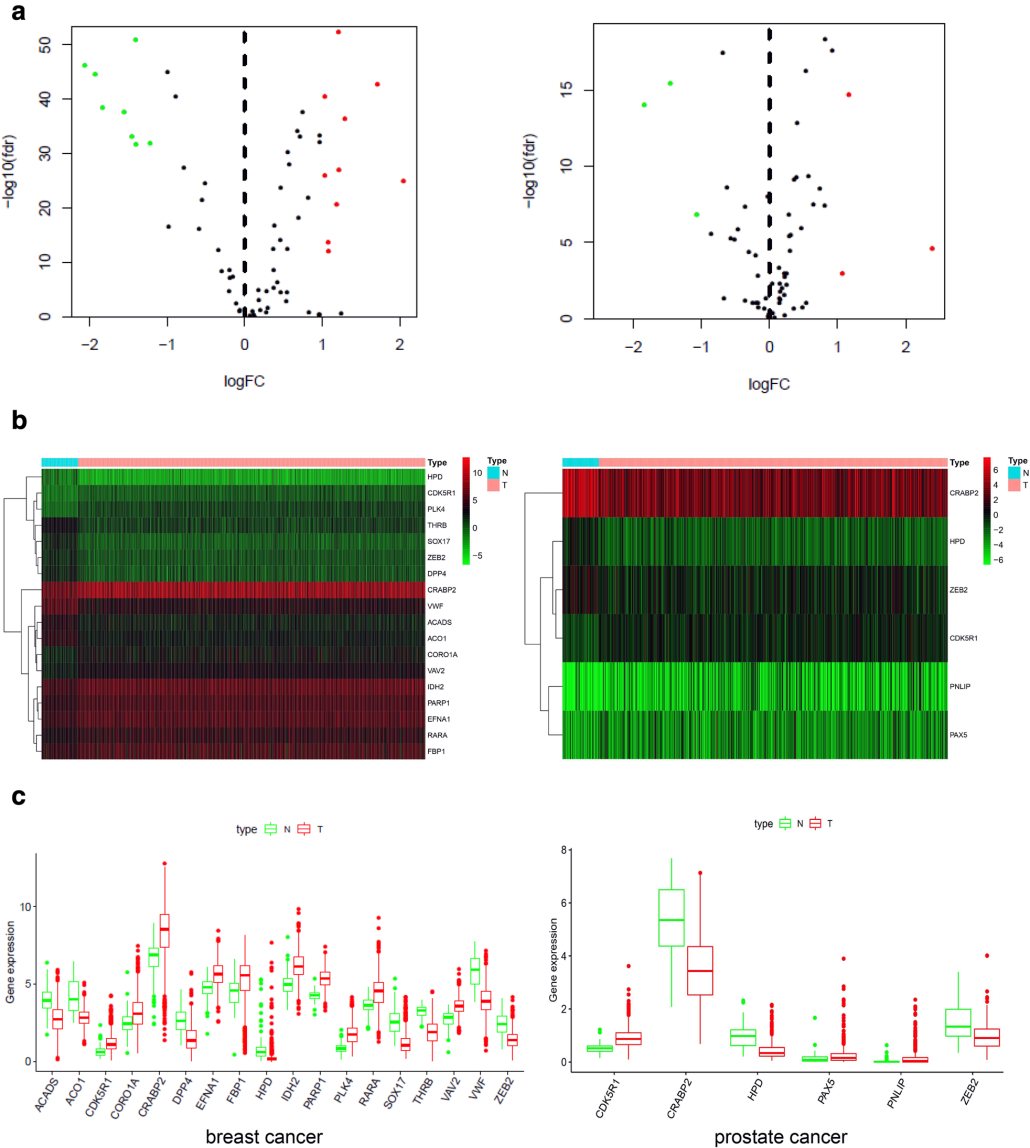
Identification of differentially expressed target genes in breast and prostate cancer. **a** Volcano plot of 76 target genes in breast and prostate cancer from TCGA database. Red plots represent aberrantly expressed mRNAs with *P* < 0.05 and absolute log FC > 1. Black plots represent normally expressed mRNAs. Green plots represent aberrantly expressed mRNAs with *P* < 0.05 and log FC < − 1. **b** Heatmap analysis of differential expression profiles between normal tissues and cancer tissues in breast and prostate. **c** Boxplot to intuitively illustrate the differences between tumor and normal tissues

We downloaded the RNA expression profiling data from TCGA database. Through a series of stringent filters was implemented, we obtained the expression levels of 76 target genes from 1164 breast samples (1053 breast tumor samples and 111 normal samples) and 551 prostate samples (499 prostate tumor samples and 52 normal samples). The genes that met the cutoff criteria of a fold change > 1 and an adjusted *P*-value < 0.05 were considered DEGs. Gene expression profiles of breast identified 18 DEGs with 10 up regulated genes and 8 down regulated genes in tumor samples when compared with normal breast tissues, and 6 DEGs with 3 up regulated genes and 3 down regulated genes (Fig. 2a). Heatmap analysis showed that these genes presented differential expression profiles between normal tissues and tumor tissues (Fig. 2b). Furthermore, DEGs were showed in boxplot to intuitively illustrate the differences between tumor and normal samples (Fig. 2c). CRABP2, HPD, ZEB2 and CDK5R1 are the common DEGs both in breast and prostate cancer.

**Fig. 3:**
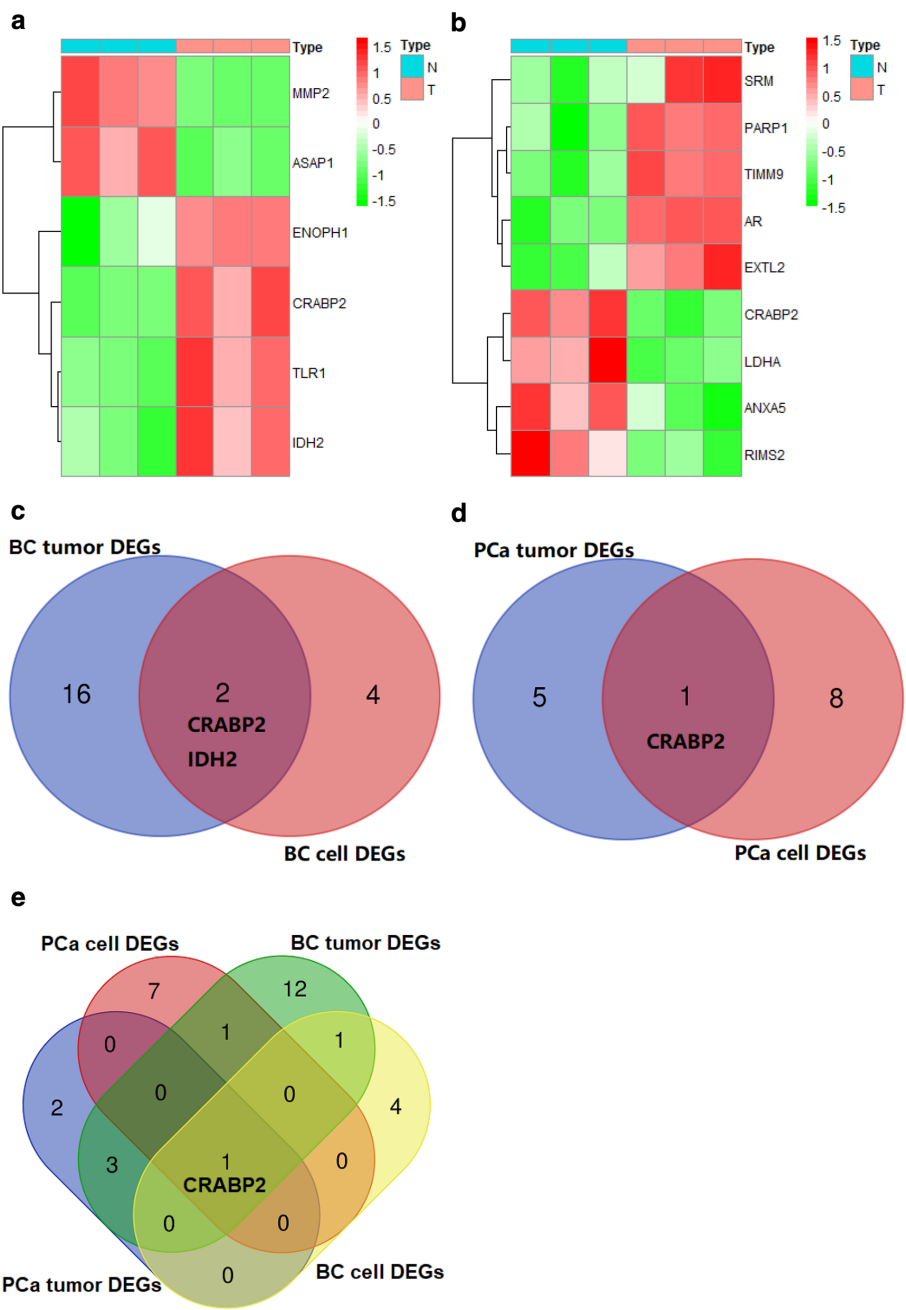
Identification of differentially expressed genes (DEGs) of goserelin in breast and prostate cells. **a** Heatmap analysis of differential expression profiles between normal breast cells (MCF-10A) and breast cancer cells (UFH-001) from GSE107209. **b** Heatmap analysis of differential expression profiles between normal prostate cells (PrEC) and prostate cancer cells (LNCaP) from GSE62410. **c** Venn diagram summarize the common DEGs both in breast cells and tissues. **d** Venn diagram summarize the common DEGs of goserelin both in prostate cells and tissues. **e** Venn diagram summarize the common DEGs both related to prostate and breast (cells and tissues)

The RNA expression profiling data of cells were downloaded from the GEO database. Heatmap analysis showed that genes have differential expression profiles between breast MCF-10A cells and 3 UFH-001 cells (Fig. 3a). A total of 6 DEGs, including 2 down regulated genes and 4 up regulated genes. Heatmap analysis showed that these genes have differential expression profiles between prostate PrEC cells and 3 LNcaP cells (Fig. 3b). A total of 9 DEGs, including 4 down regulated genes and 5 up regulated genes. The results of venn analysis showed that CRABP2 and IDH2 were the common DEGs both in breast cells and tissues (Fig. 3c). CRABP2 was the common DEGs in prostate cells and tissues (Fig. 3d). The results indicate that CRABP2 is the significantly DEGs related to prostate and breast cancers (Fig. 3e).

**Fig. 4:**
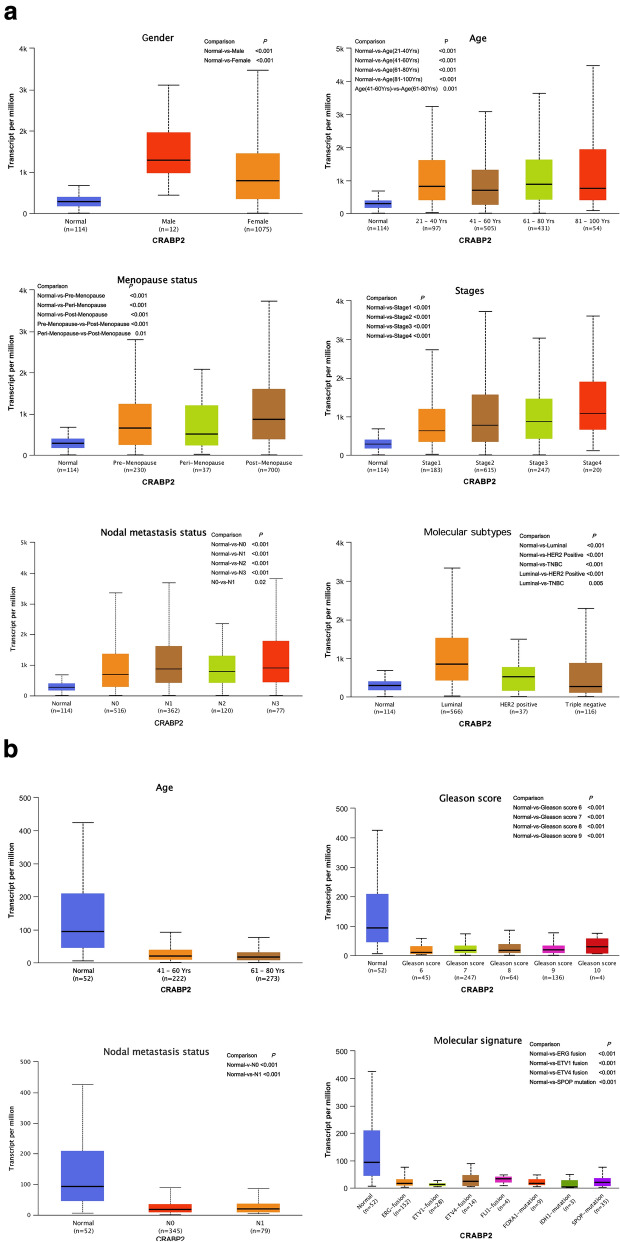
Conducting clinical correlation analysis of CRABP2 by UALCAN. **a** CRABP2 transcription in subgroups of patients with breast cancer, stratified based on gender, age menopause status, disease stage, nodal metastasis status, and molecular subtypes. **b** CRABP2 transcription in subgroups of patients with prostate cancer, stratified based on age, gleason score, nodal metastasis status, and molecular signature

The expression of CRABP2 was significantly higher in breast cancer than normal controls in subgroup analysis based on gender, age, menopause status, disease stage, nodal metastasis status, and molecular subtypes (all *P* < 0.05, Fig. 4a). We also compared the relative expression levels of CRABP2 between subgroups in breast cancer tissues, and found that post-menopause was higher than per-menopause (*P* < 0.001) and peri-menopause (*P* = 0.01), N1 was higher than N0 (*P* = 0.02), luminal subtype was higher than triple negative breast cancer (TNBC) (*P* = 0.005) and HER-2 positive subtype (*P* < 0.001). However, the expression levels of CRABP2 based on gleason score group, nodal metastasis group, and molecular signature group in prostate cancer were lower than normal controls (all *P* < 0.05, Fig. 4b).

### Survival analysis of target genes of goserelin in TCGA data

**Fig. 5:**
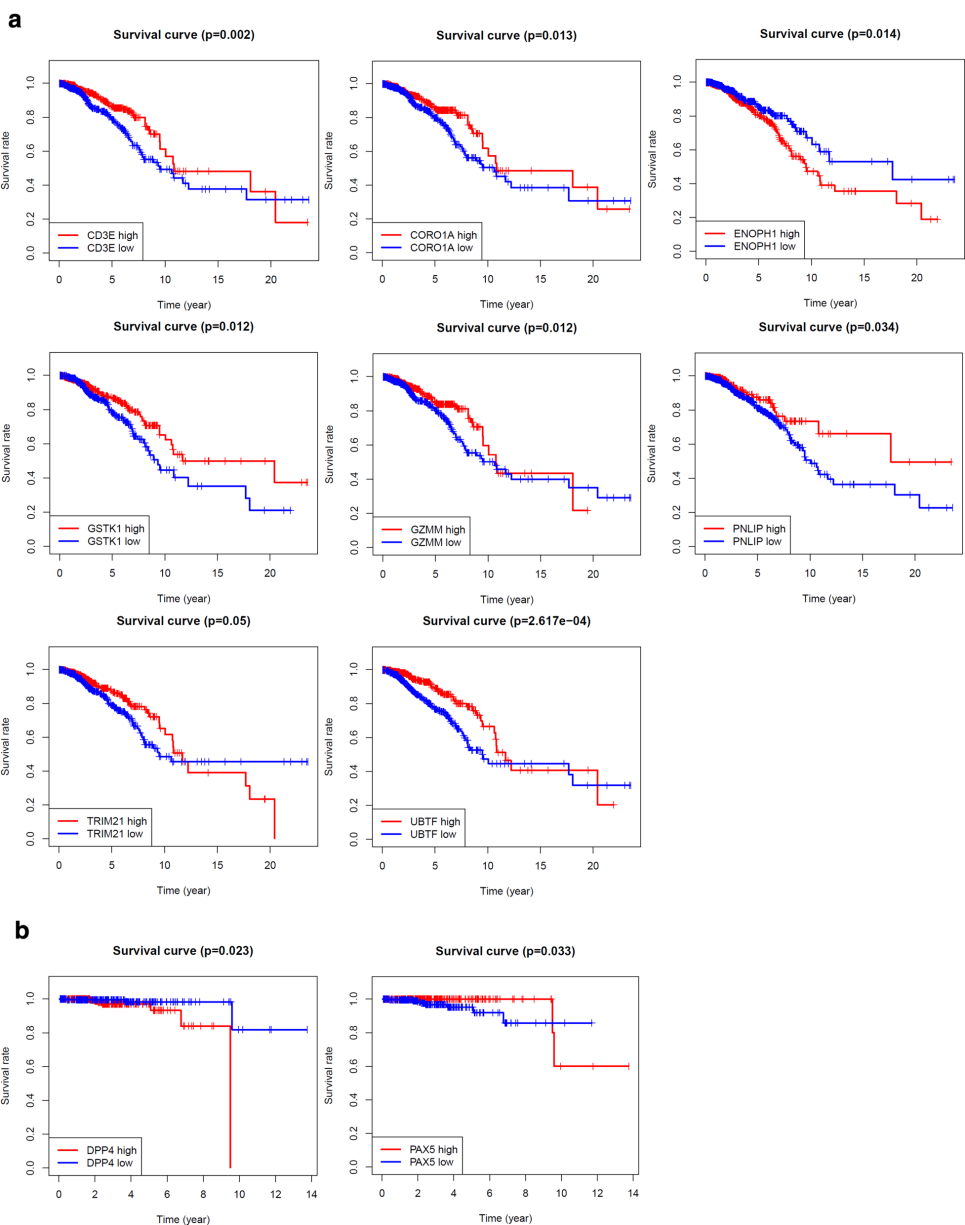
Prognostic value of goserelin target genes in TCGA. **a** Kaplan–Meier overall survival curve for breast cancer patients with high and low indicated gene expression. **b** Kaplan–Meier overall survival curve for prostate cancer patients with high and low indicated gene expression

Kaplan–Meier survival analysis was performed based on TCGA survival data, and only log-rank P value < 0.05 was shown in Fig. 5a, b. Genes expression of CD3E, CORO1A, ENOPH1, GSTK1, GZMM, PNLIP, TRIM21 and UBTF are significantly related to the prognosis of breast cancer patients (Fig. 5a). DPP4 and PAX5 are significantly associated with the prognosis of prostate cancer patients (Fig. 5b).

### Constructing and evaluating the breast and prostate cancer risk prediction model by target genes of goserelin

**Fig. 6:**
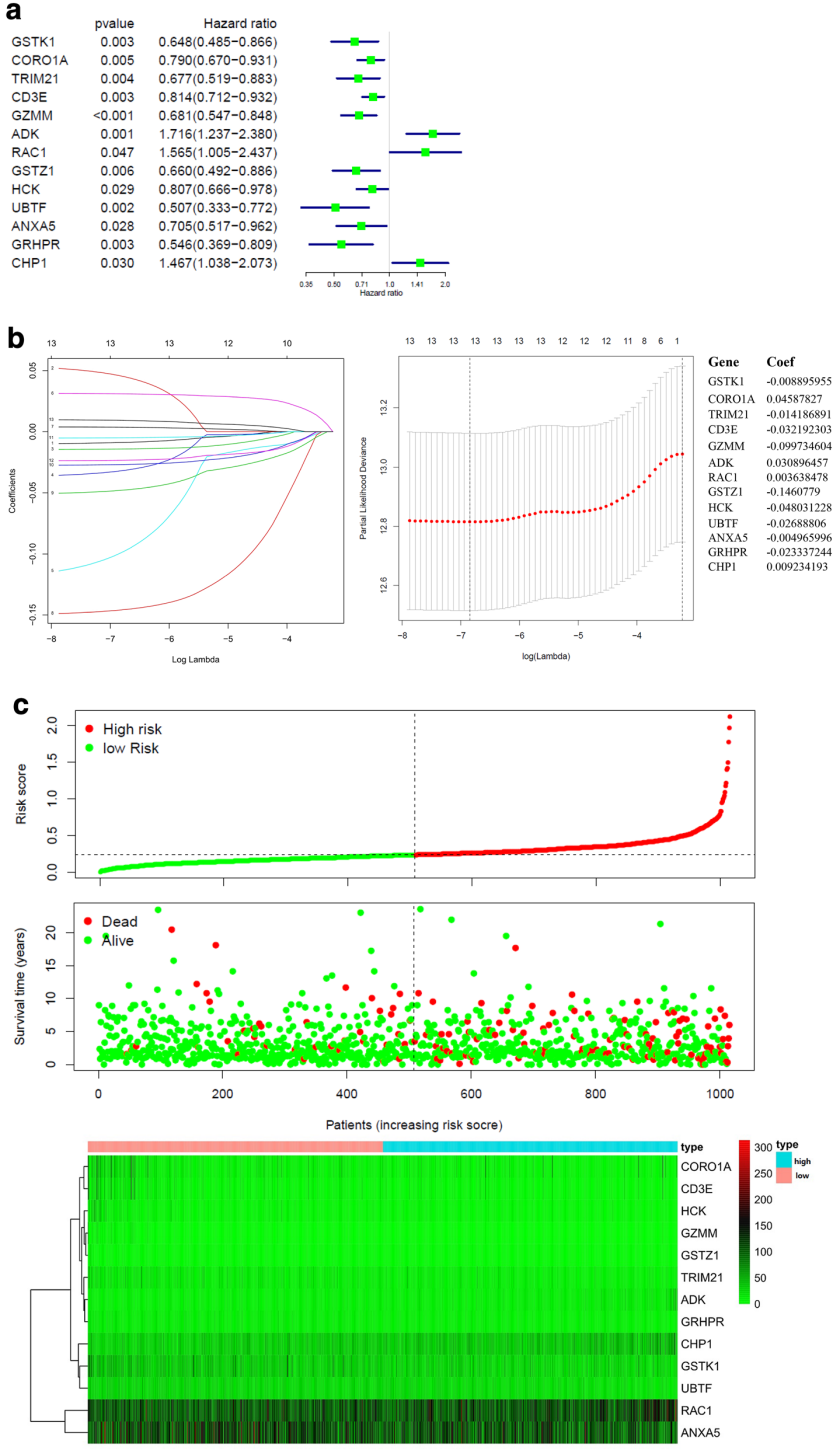

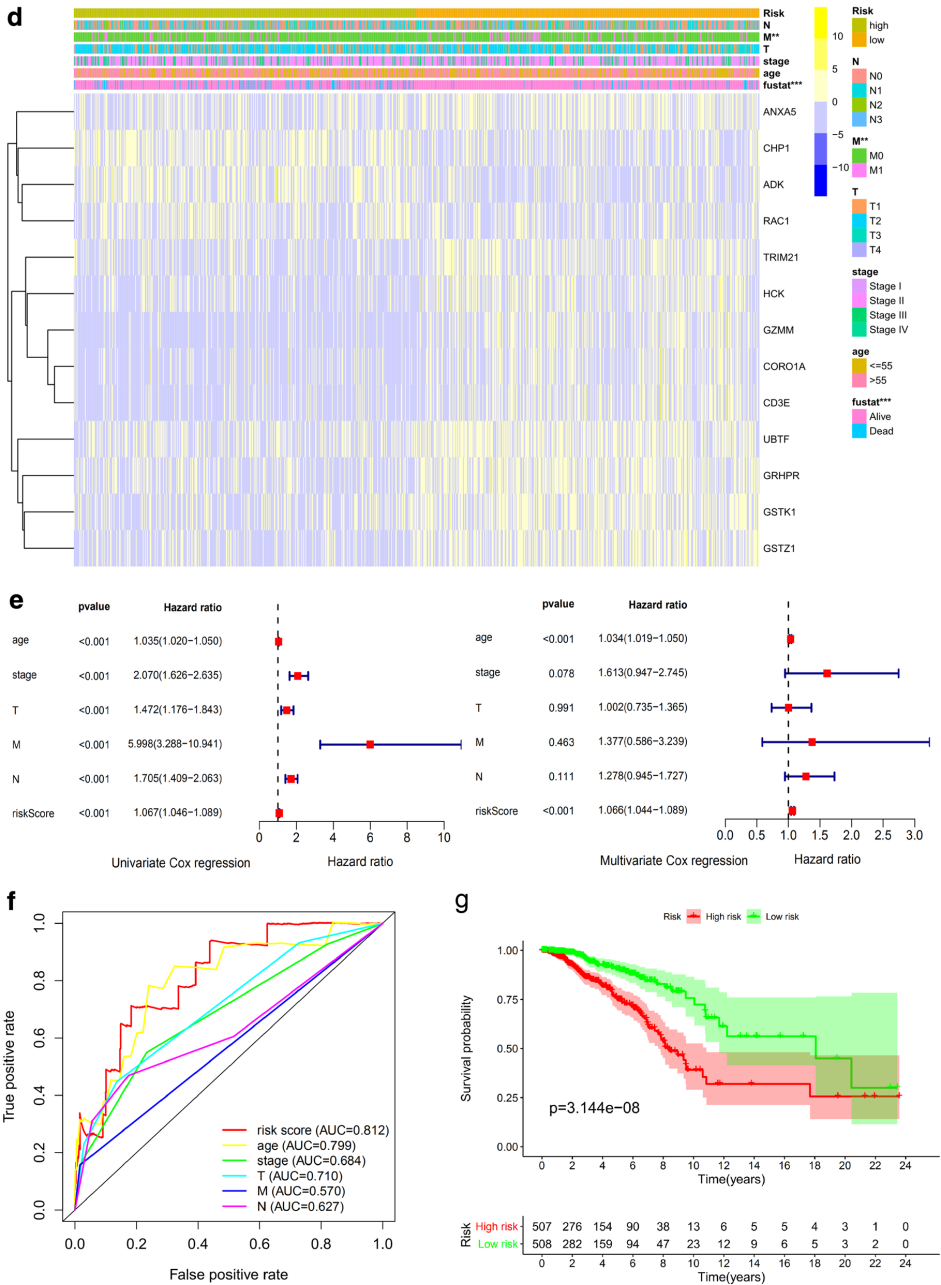

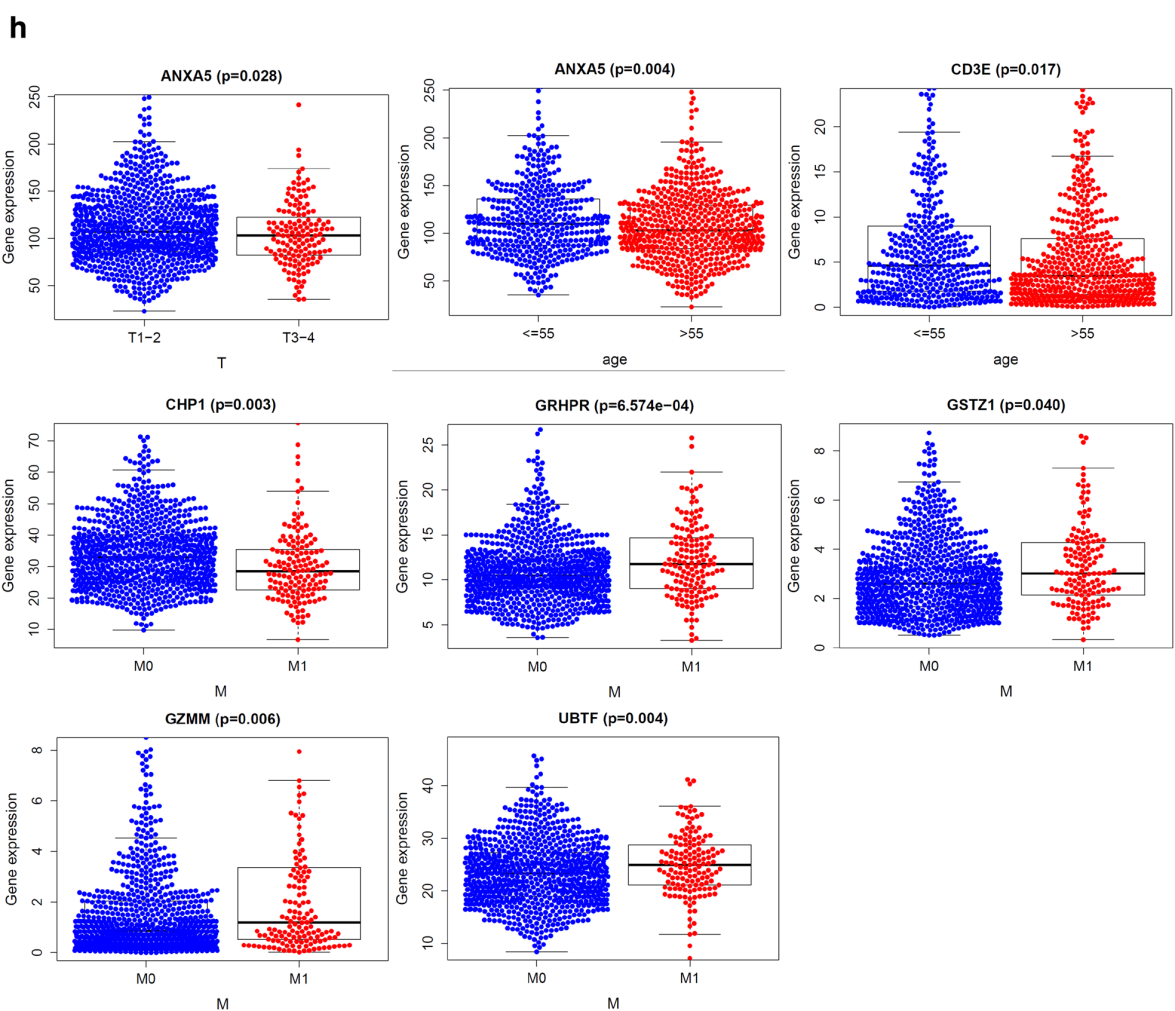
Construction and evaluation of the breast risk prediction model in TCGA. **a** Overall survival in univariate Cox regression of target genes. **b** Lasso regression for genes in univariate Cox regression. **c** The horizontal axis represents the samples, and the vertical axis represents risk scores (top), overall survival (middle), and target genes (bottom). **d** The heatmap shows the expression of the 13 genes in high-risk and low-risk groups. The distribution of clinic-pathological characteristics was compared between the high-risk and low-risk groups. *P < 0.05, **P < 0.01 and ***P < 0.001. **e** Univariate and multivariate Cox regression analysis of the clinic-pathological factors (including risk score) related to overall survival. **f** ROC curves showed the predictive efficiency of the clinic-pathological factors (including risk score). **g** Kaplan–Meier overall survival curve for patients with high-risk group and low-risk group. **h** The expression of genes in lasso regression related to clinic-pathological factors

**Fig. 7:**
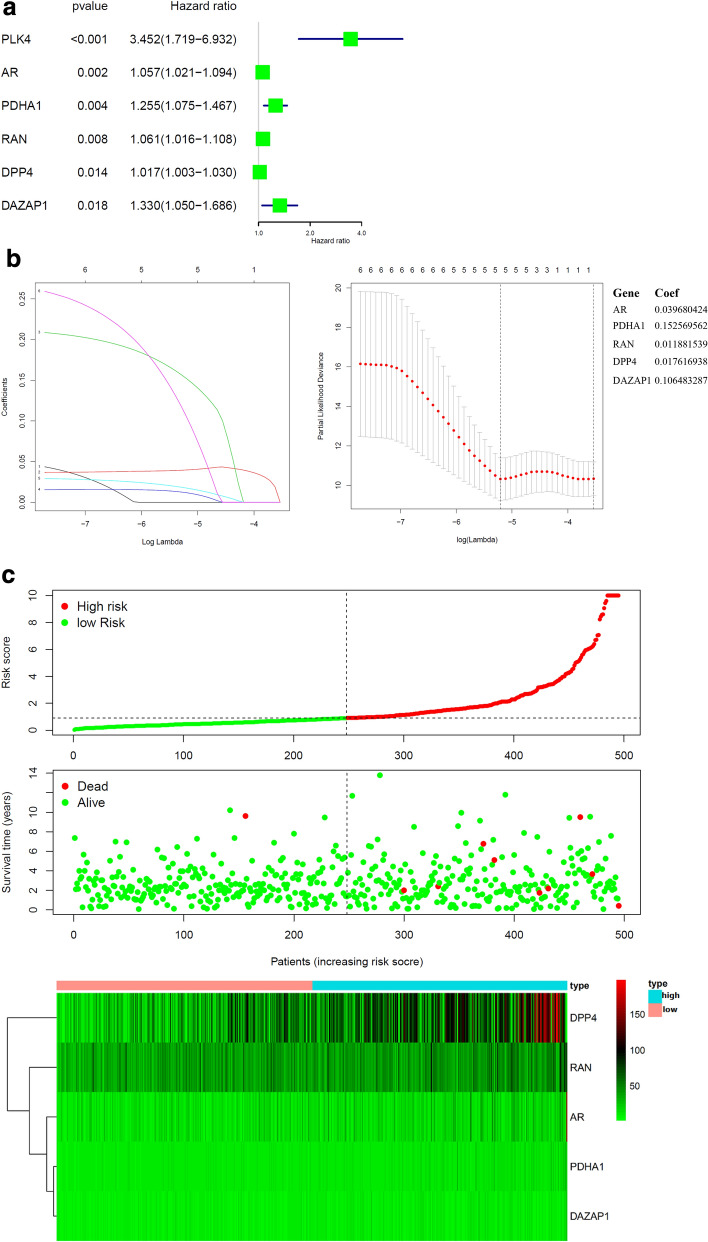

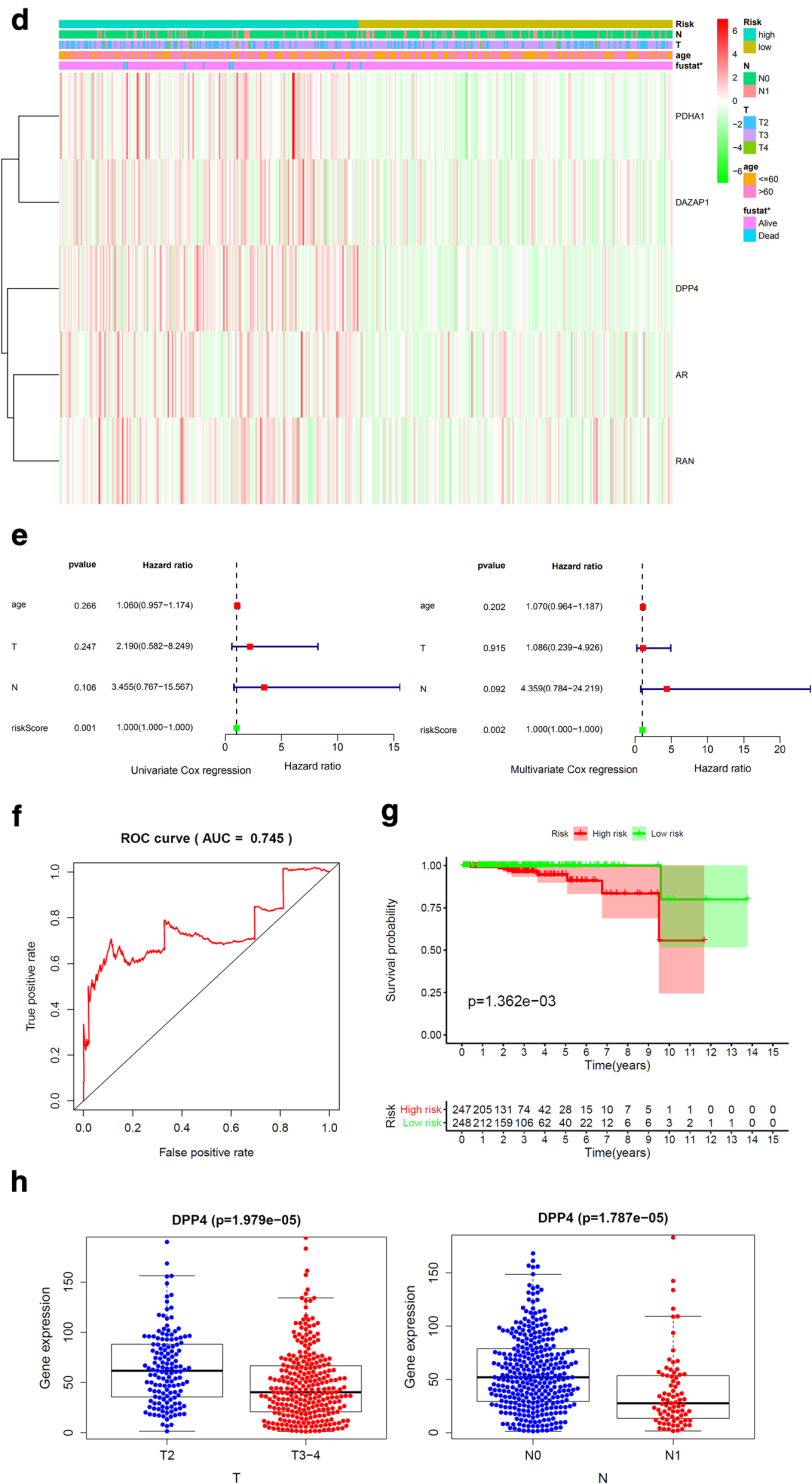
Construction and evaluation of the prostate cancer risk prediction model in TCGA. **a** Overall survival in univariate Cox regression of target genes. **b** Lasso regression for genes in univariate Cox regression. **c** The horizontal axis represents the samples, and the vertical axis represents risk scores (top), overall survival (middle), and target genes (bottom). **d** The heatmap shows the expression of the 5 genes in high-risk and low-risk groups. The distribution of clinic-pathological characteristics was compared between the high-risk and low-risk groups. *P < 0.05, **P < 0.01 and ***P < 0.001. **e** Univariate and multivariate Cox regression analysis of the clinic-pathological factors (including risk score) related to overall survival. **f** ROC curve showed the predictive efficiency of the risk score. **g** Kaplan–Meier overall survival curve for patients with high-risk group and low-risk group. **h** The expression of genes in lasso regression related to clinic-pathological factors

In order to investigate the prognostic value of these 76 target genes in breast and prostate cancer, respectively. Univariate Cox survival regression analysis was performed based on the expression levels of the genes from TCGA (Figs. 4a and 7a). We selected the survival associated genes as candidates to create a lasso regression model. Eventually, the risk prediction model of breast cancer with 13 genes, including GSTK1, CORO1A, TRIM21, CD3E, GZMM, ADK, RAC1, GSTZ1, HCK, UBTF, ANXA5, GRHPR, CHP1, was generated and the coefficient of each independent prognostic gene was shown in Fig. 4b. The lasso results also showed that five genes (AR, PDHA1, RAN, DPP4 and DAZAP1) were the powerful composed factors of prostate cancer risk prediction model (Fig. 7b). All samples were divided into two (high and low) groups according to the median value of risk score of our model. Both in breast and prostate cancer model, the heatmap of composed genes expression were shown in Figs. 6c and 7c, and the samples of deaths was significantly higher in the high risk group compared to the low risk group (Figs. 6c and 7c). The expression levels of the genes in high-risk and low-risk group were presented in the heatmap with clinicopathologic variables as the annotations (Figs. 6d and 7d). In the breast cancer risk prediction model, the results showed that there were significant differences between the high risk and low risk groups in term of survival (*P* < 0.001), and stage M (*P* < 0.05). While survival status (*P* < 0.05) was the only difference between high risk and low risk groups in the prostate cancer risk model.

To test whether the risk signature was an independent prognostic factor, univariate and multivariate Cox regression analyses were performed. As a result, the age at diagnosis, pathological stage, stage TNM and risk score were associated with OS (all *P* < 0.001) of breast cancer patients in univariate analysis and only risk score and age at diagnosis were still significantly related to OS (all *P* < 0.001) in multivariate Cox regression analysis(Fig. 6e), while only the risk score was significantly related to OS (all *P* < 0.05) of prostate cancer patients in both univariate and multivariate Cox regression analysis (Fig. 7e). Furthermore, time-dependent ROC curve showed that the risk score (AUC = 0.812) was better than other factors in predicting for OS of breast cancer patients (Fig. 6f). In prostate cancer patients, the ROC curve was used to assess the sensitivity and specificity of the prediction and the result showed that AUC values was 0.745, suggesting well-prediction performances (Fig. 7f). The result of Kaplan–Meier survival analysis both in breast and prostate cancer patients showed that the high-risk group had significantly shorter survival time compared to low-risk group (Figs. 6g and 7g). Among the 13 genes that was built of the breast cancer risk prediction model, the expression levels of ANXA5 were related to stage T (*P* = 0.028) and age (*P* = 0.004), CD3E expression levels (*P* = 0.017) were only related to age, GSTZ1 (*P* = 0.040), GZMM (*P* = 0.006), GRHPR (*P* < 0.001), UBTF (*P* = 0.004) and CHP1 (*P* = 0.003) were significantly related to stage M (Fig. 6h). Among the 5 genes that was built of the prostate cancer risk prediction model, the expression levels of DPP4 were related to stage T (*P* < 0.001) and stage N (*P* < 0.001), (Fig. 7h).

### Generating novel prognosis subgroups and their clinical intercluster prognosis analysis

**Fig. 8:**
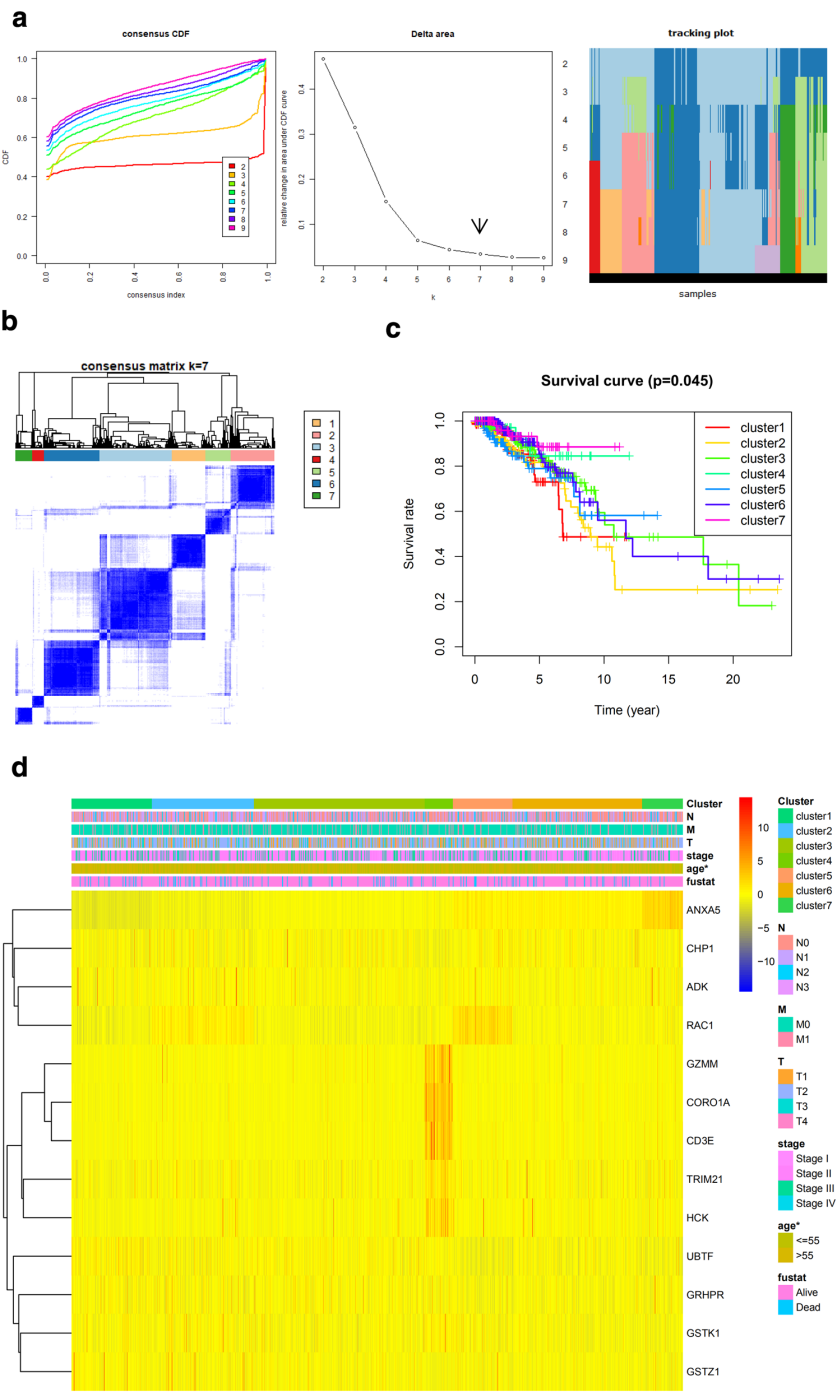
Defining breast cancer signature by consensus clustering and evaluating its prognostic value. **a** Criteria for selecting number of categories. **b** Color-coded heatmap corresponding to the consensus matrix for k = 7 obtained by applying consensus clustering. Color gradients represent consensus values from 0–1; white corresponds to 0 and dark blue to 1. **c** Kaplan–Meier plot showing the OS for the seven classes. **d** The expression levels of the genes in different clusters were presented in the heatmap with clinic-pathological variables as the annotations

**Fig. 9:**
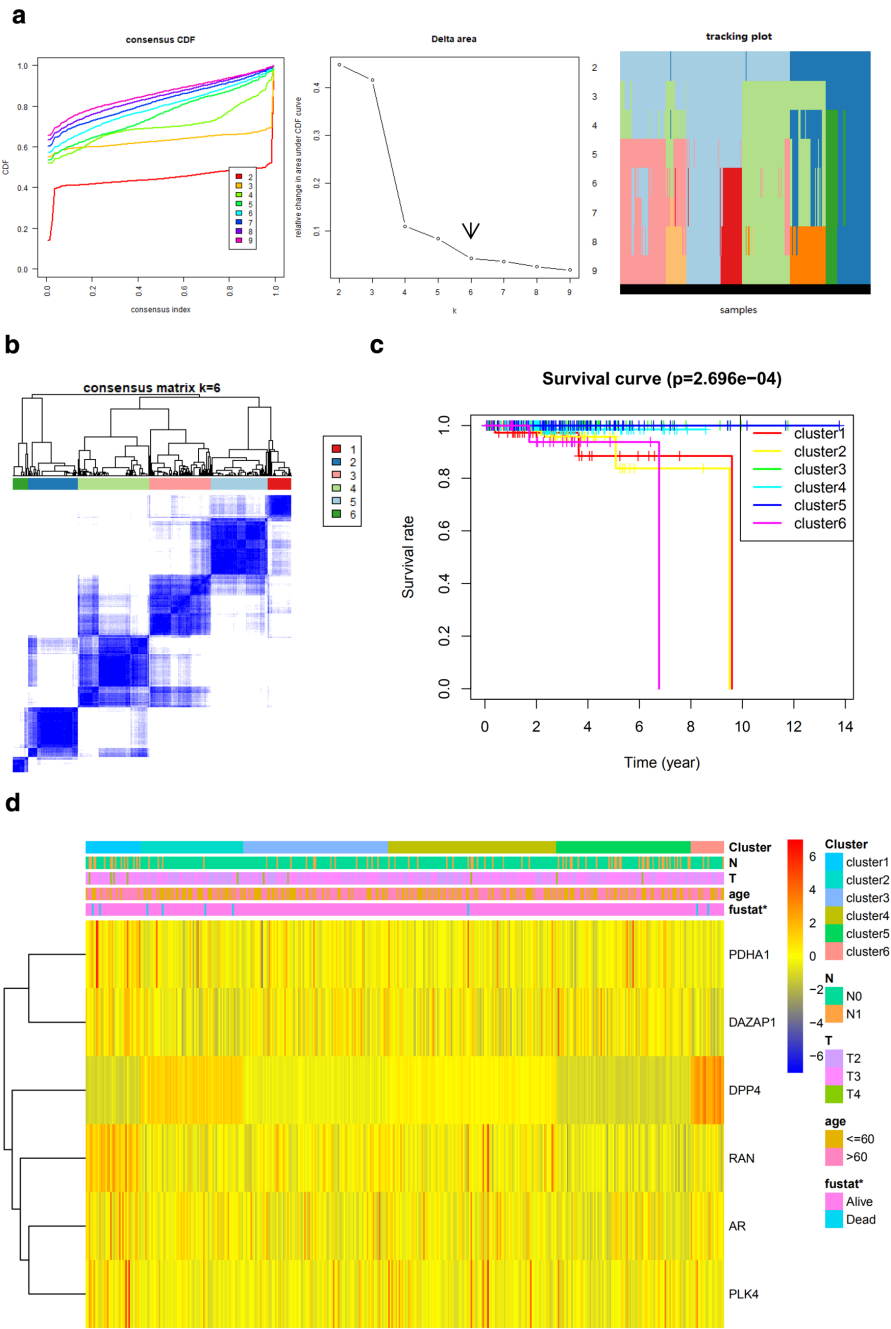
Defining prostate cancer signature by consensus clustering and evaluating its prognostic value. **a** Criteria for selecting number of categories. **b** Color-coded heatmap corresponding to the consensus matrix for k = 6 obtained by applying consensus clustering. Color gradients represent consensus values from 0–1; white corresponds to 0 and dark blue to 1. **c** Kaplan–Meier plot showing the OS for the six classes. **d** The expression levels of the genes in different clusters were presented in the heatmap with clinic-pathological variables as the annotations

We selected the survival associated genes from univariate Cox survival regression analysis as candidates to perform consensus clustering. As a result, 13 potential prognostic genes was used to identify subgroups of breast cancer for prognostic purposes, and 6 genes was used to identify subgroups of prostate cancer. Numbers of clusters were determined according to the following criteria: relatively high consistency within the cluster, relatively low coefficient of variation, and no appreciable increase in the area under the Cumulative Distribution Function (CDF) curve. We calculated average cluster consensus and the coefficient of variation among clusters depending on category number (Figs. 8a and 9a). To improve the prognostic value of the COAD classifications, we choose larger cluster numbers when possible. Hence, when k = 7 (Fig. 8b) in breast cancer and k = 6 (Fig. 9b) in prostate cancer could be the optimal choice with clustering increasing from k = 2–9. Kaplan–Meier survival analysis of breast cancer revealed significant differences in prognosis among the 7 clusters (*P* < 0.05), cluster 7 had the best prognoses, while cluster 2 had the worst (Fig. 8c). Kaplan–Meier survival analysis of prostate cancer revealed significant differences in prognosis among the 6 clusters (*P* < 0.001), cluster 5 had the best prognoses, while cluster 1 had the worst (Fig. 9c). The expression levels of the genes in different clusters were presented in the heatmap with clinic-pathological variables as the annotations (Figs. 8d and 9d). The results showed that there were significant differences between the 7 breast cancer clusters in term of age (*P* < 0.05, Fig. 8d), and survival status was difference among the 6 prostate cancer clusters (*P* < 0.05, Fig. 9d).

### Risk prediction model was validated in very young breast cancer patients use ICGC data

Women younger than 35 years are more demanding for fertility. Goserelin is the most commonly ovarian suppression strategies among premenopausal women with hormone positive breast cancer owing mainly to its non-invasiveness and reversibility [15]. The gene expression data and clinical data of 50 breast cancer patients younger than 35 years were downloaded from ICGC portal. Univariate Cox survival regression analysis (OS and RFS) was performed to investigate the candidates to create a lasso regression model in very young breast cancer patients by ICGC data (Additional file 1: Figure S2a, S2b). Eventually, the risk prediction model of OS with 5 genes, including HCK, ASAP1, NDST1, EXTL2, MSX1, was generated and the coefficient of each independent prognostic gene was shown in Additional file 1: Figure S2c. The lasso results also showed that seven genes (AR, APPL1, ASAP1, NDST1, GRHPR, PPP2R1A, and TRIM21) were the powerful composed factors of RFS risk prediction model (Additional file 1: Figure S2d). The result of survival analysis (both in OS and RFS) showed that the high-risk group had significantly worse prognosis compared to low-risk group (Additional file 1: Figure S2e). The models performed well as the AUCs were equal to 0.908 at OS and 0.998 at RFS (Additional file 1: Figure S2f). The results showed that the model of very young breast cancer patients we constructed exhibited good classifier performance. ASAP1 and NDST1 are the common genes that build risk prediction models for OS and RFS.

### OS and RFS analysis of very young breast cancer patients in ICGC data

**Fig. 10:**
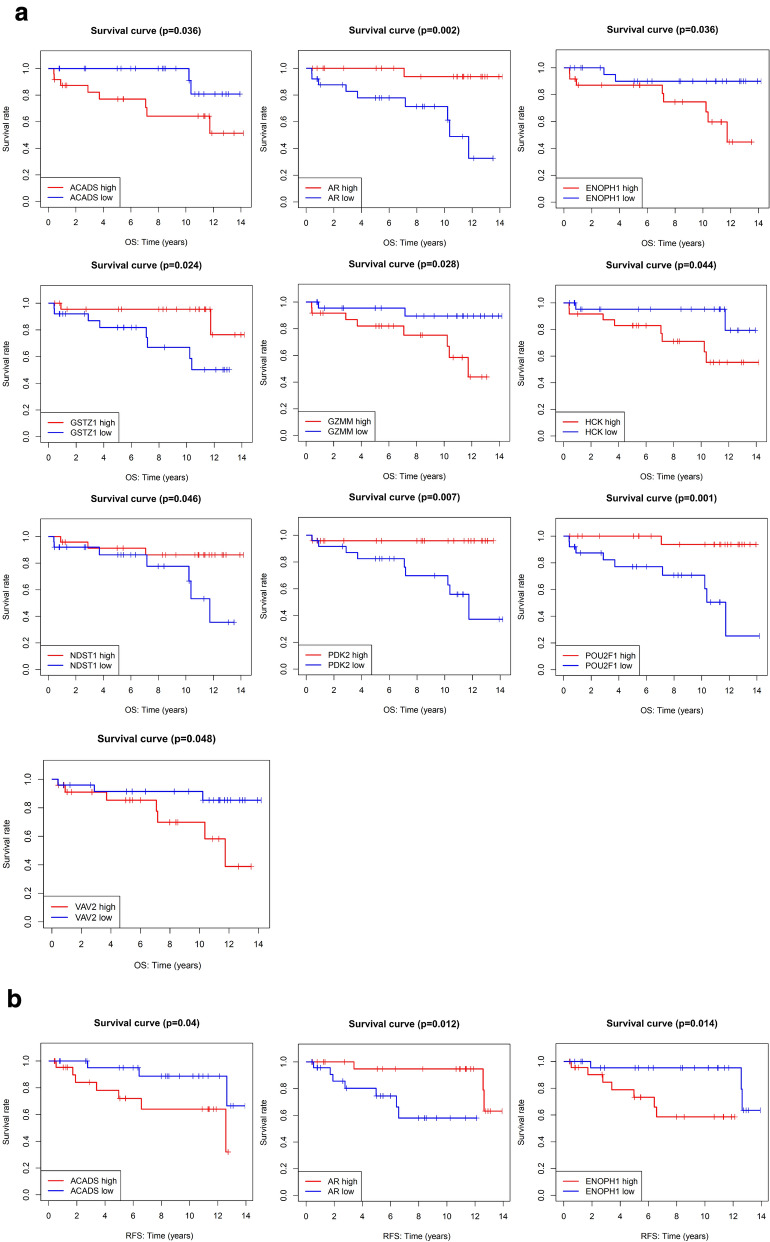

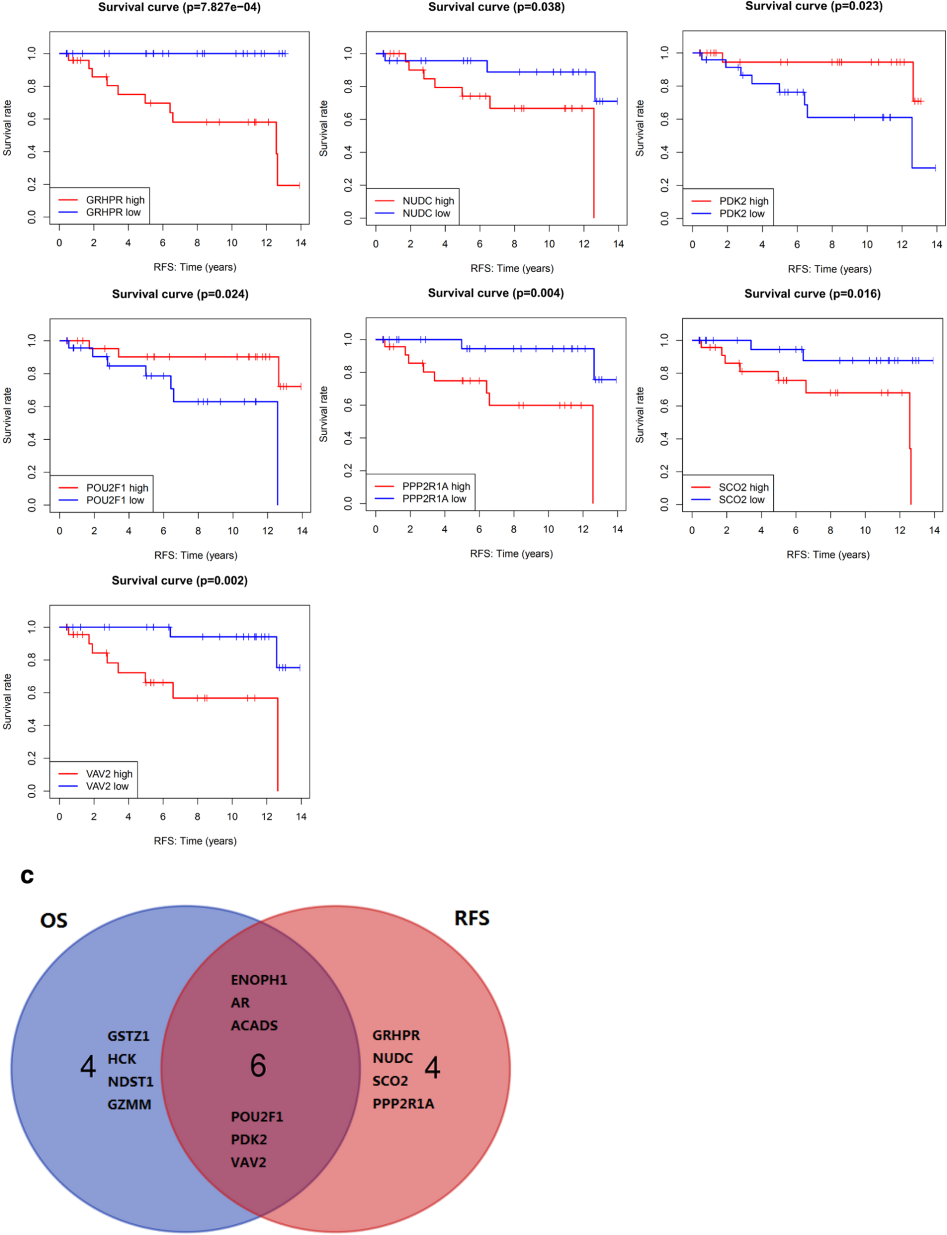
The prognostic value of goserelin target genes in breast cancer patients younger than 35 years in ICGC data. **a** Kaplan–Meier overall survival curve for patients with high and low indicated gene expression. **b** Kaplan–Meier recurrence-free survival curve for patients with high and low indicated gene expression. **c** Venn diagram summarize the representative target genes both related to OS and RFS

Kaplan–Meier survival analysis of OS and RFS were performed based on ICGC survival data, and only log-rank *P* value < 0.05 was shown in Fig. 10a, b. Genes expression of POU2F1, AR, PDK2, GSTZ1, GZMM, ACADS, ENOPH1, HCK, NDST1, and VAV2 are significantly related to the OS of breast cancer patients younger than 35 years (Fig. 10a), and POU2F1, AR, PDK2, GRHPR, SCO2, ACADS, ENOPH1, PPP2R1A, NUDC, and VAV2 are significantly associated with the RFS (Fig. 10b). The venn results also showed that six genes (POU2F1, AR, PDK2, ACADS, ENOPH1, and VAV2) were both related to OS and RFS in Kaplan–Meier survival analysis (Fig. 10c).

### Screening representative target gene and conducting clinical correlation analysis

**Fig. 11:**
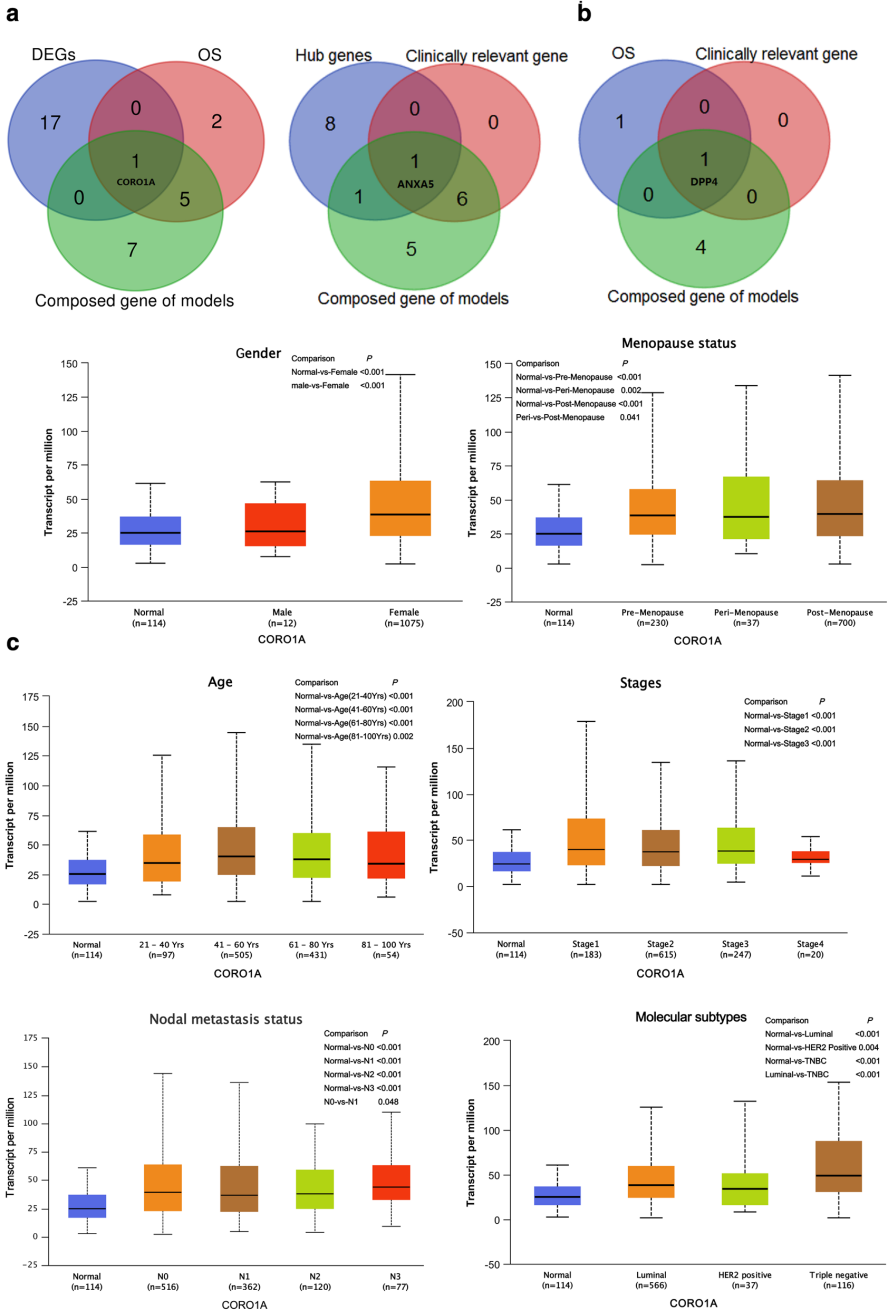

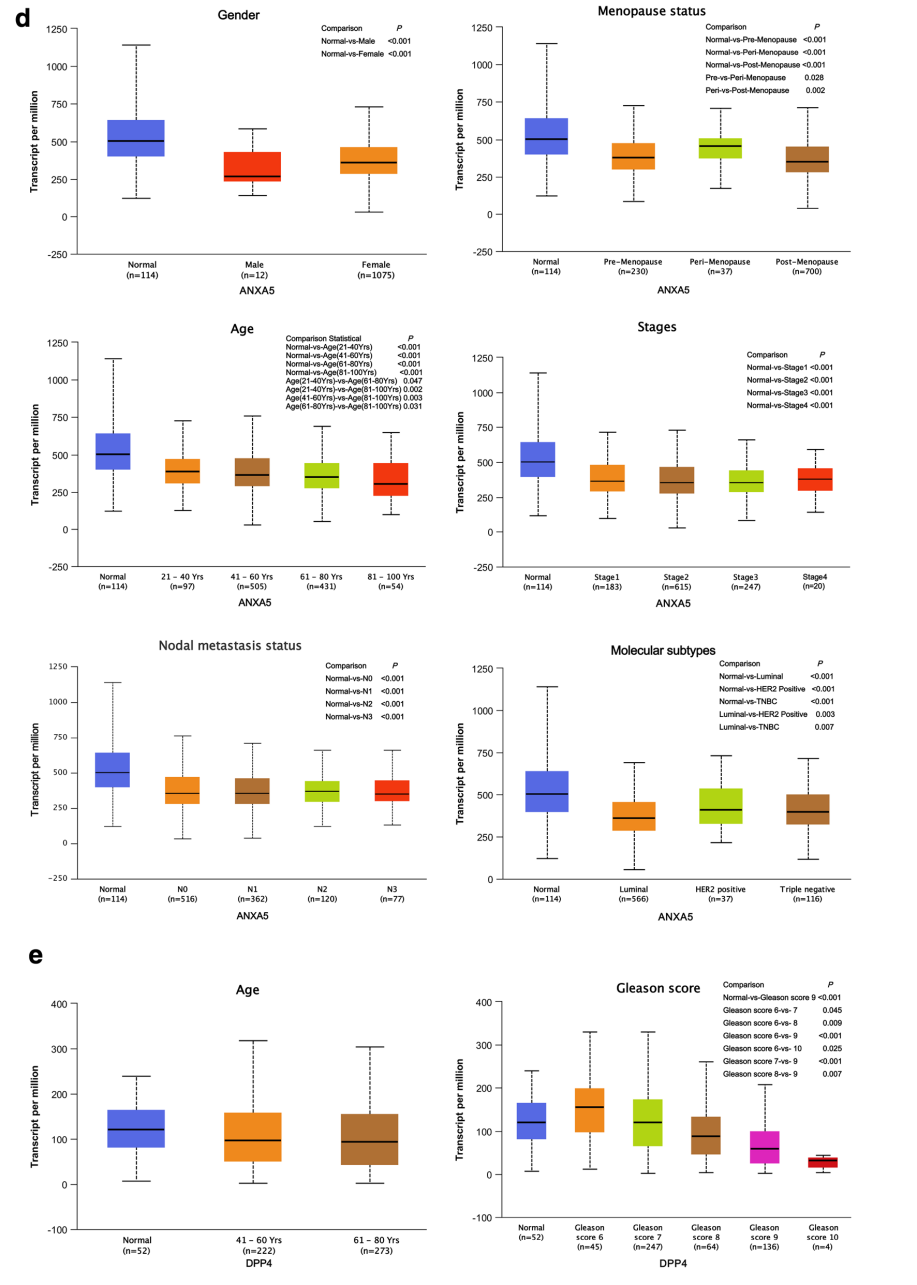

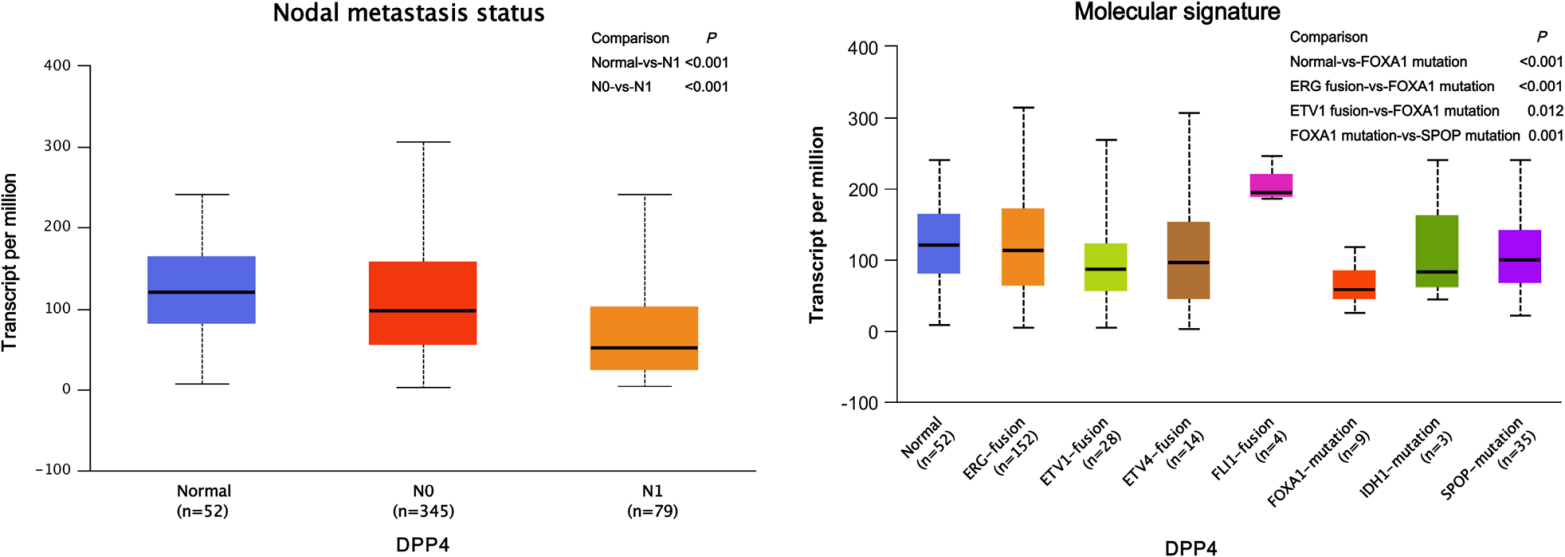
Screening representative target gene and conducting clinical correlation analysis (UALCAN). **a** Venn diagram summarize the representative target genes of breast cancer, CORO1A screening in left and ANXA5 in right. **b** Venn diagram summarize the representative target genes of prostate cancer. **c** CORO1A transcription in subgroups of patients with breast cancer, stratified based on gender, age menopause status, disease stage, nodal metastasis status, and molecular subtypes. **d** ANXA5 transcription in subgroups of patients with breast cancer, stratified based on gender, age menopause status, disease stage, nodal metastasis status, and molecular subtypes. **e** DPP4 transcription in subgroups of patients with prostate cancer, stratified based on age, gleason score, nodal metastasis status, and molecular signature

Venn diagram summarize amount of predicted composed gene of risk prediction models links for DEGs, OS, hub genes or clinical factors. Eventually, CORO1A and ANXA5 as the representative target genes for breast cancer (Fig. 11a) and DPP4 for prostate cancer (Fig. 11b) were made the following analysis. The results of the boxplot indicated that there were significant differential expression of three representative target genes between male and female in normal breast tissues (Additional file 1: Figure S3). Additional file 1: Table S1 shows the results of the GO enrichment analysis of BP category. CORO1A was mainly enriched in 15 BP items, and 3 among them (including positive regulation of T cell activation, positive regulation of leukocyte cell–cell adhesion and positive regulation of cell–cell adhesion) were co-enriched with DPP4. ANXA5 was mainly enriched in 3 BP items, and peptide hormone secretion was co-enriched with DPP4. Thus, CORO1A, DPP4 and ANXA5 might play the significant roles in some common biological process of tumor.

We assessed the expression levels of CORO1A and ANXA5 in breast samples by subgroup analysis of multiple clinic-pathological features. The expression of CORO1A was significantly higher in breast cancer patients than normal controls in subgroup analysis based on female, age, menopause status, disease stage 1–3, nodal metastasis status, and molecular subtypes (all *P* < 0.05, Fig. 11c). We also compared the relative expression levels of CORO1A between subgroups in breast cancer tissues, and found that female was higher than male (*P* < 0.001), post-menopause was higher than peri-menopause (*P* = 0.041), N1 was higher than N0 (*P* = 0.048), triple negative breast cancer (TNBC) was higher than luminal subtype (*P* < 0.001). The expression of ANXA5 was significantly lower in breast cancer patients than normal controls in subgroup analysis based on gender, age, menopause status, disease stage, nodal metastasis status, and molecular subtypes (all *P* < 0.001, Fig. 11d). We also compared the relative expression levels of ANXA5 between subgroups in breast cancer tissues, and found that luminal subtype was lower than TNBC (*P* = 0.007) and HER2 postitive (*P* = 0.003) subtypes, peri-menopause was higher than per-menopause (*P* = 0.028) and post-menopause (*P* = 0.002), and as the age progressed, the expression of ANXA5 decreased (all *P* < 0.05, Fig. 11d). The expression levels of DPP4 based on gleason score 9 group, N1 nodal metastasis group, and FOXA1 mutation group in prostate cancer tissues were lower than normal controls (all *P* < 0.001, Fig. 11e). We then assessed the expression of DPP4 between subgroups in prostate cancer tissues, and found DPP4 expression in N1 was low than N0 (P < 0.001). As the gleason score increased, the expression of DPP4 decreased. The expression of DPP4 was lowest in FOXA1 mutation group, and found a statistically significant only compared to ETV1 fusion (*P* = 0.012), ERG fusion (*P* < 0.001), SPOP mutation (*P* = 0.001) groups (Fig. 11e). Thus, the expression of CORO1A and ANXA5 may serve as the potential diagnostic indicators in breast cancer, and DPP4 might play a significant role in the tumorigenesis and progression of prostate cancer.

## Discussion

Goserelin is an accepted alternative to diethylstilbestrol or orchidectomy in the management of men with prostate cancer. Goserelin was the most widely accepted upfront method of ovarian suppression in premenopausal women (especially for women who want to have children) due to its advantage of being easy to administer, noninvasive and reversible. As palliative treatment for premenopausal women with ER-positive breast cancer, goserelin may similarly rival ovariectomy [16]. Androgen-deprivation therapy (ADT) has traditionally been the mainstay of patients diagnosed with advanced prostate cancer [17]. Because treatment of advanced prostate cancer is only palliative, treatment should be aimed at enhancing quality of life, and treatment with goserelin has a more favourable effect on the quality of life than surgical castration. However, not all users of goserelin can benefit from it, or some patients are not sensitive to goserelin. The advent of network pharmacology has highlighted the need for accurate treatment and predictive biomarkers. Network pharmacology approaches have been used from two points of view: (1) to identify novel targets and (2) to determine which unknown signaling pathways interact with compounds [18]. In this context, disease enrichment analysis indicates that goserelin might be related with cancer pathways in general, suggesting its potential as an antitumoral compound. Our data showed that the compound of goserelin yielded 76 candidate target genes. In order to improve the treatment management of goserelin, it is important to identify novel drug targets that can improve prognostic evaluation, recurrence prediction, and the success of medication. However, few study has demonstrated that drug targets can be used for construction of disease subgroups and prognostic evaluation.

In the present study, we identified 18 DEGs in breast cancer tissues and 5 DEGs in cells, and 6 DEGs in prostate cancer tissues and 9 DEGs in cells. CRABP2 is the common DEG both in breast and prostate cancer. Previous reports state that abnormal expression of CRABP2 is associated with malignant cancers in the human being [19]. In our study, CRABP2 is highly expressed in breast cancer, and lowly expressed in prostate cancer. CRABP2 transports RA to the retinoic acid receptor (RAR) in the nucleus and regulates cell proliferation, apoptosis, invasion, and metastasis. However, how RA regulates CRABP2 in mammary cancer invasion and metastasis requires further investigation. Murphy et al. [20] used multiple omics platforms to integrate biomarkers to improve the stratification of patients with aggressive and indolent prostate cancer. This study is of high research value, but its risk stratification of the disease depends on multiple sets of data, which may increase the complexity and cost of diagnosis. Considering the experiences from a previous study showed that a combination of multiple genes manifests more efficiently than a single gene as a diagnostic or prognostic biomarker [21]. Next, univariate Cox regression followed by lasso regression was used to validate independent factors and construct a risk prediction model for breast and prostate cancer patients in our study. Although the composed genes of the risk prediction models for breast and prostate cancer were completely different, the low-risk group had significantly better survival than the high-risk group in both models. The results of ROC also showed that the model we constructed exhibited good classifier performance. Although the well predictive value of model was available, the significance of these genes in relation to tumor classification, survival time, and prognosis need to be confirmed in more groups of patients. Then, seven breast cancer subgroups (cluster1- cluster7) and six prostate cancer subgroups (cluster1- cluster6) were identified by consensus clustering according to the expressions of the target genes, which were selected for construction of the risk signature. We found that there were significant statistical differences in survival between the clusters of both breast and prostate cancer. Therefore, the candidate target genes of goserelin may serve as the potential prognosis indicators screened by univariate Cox regression analysis.

On the basis of various prospective and retrospective studies of breast cancer, age is an independent prognostic factor with worse survival [22–26]. Recent reports from large clinical trials have established that tamoxifen or aromatase inhibitor (AI) in combination with ovarian suppression strategies was superior to tamoxifen alone in very young women [27, 28]. Medical ovarian suppression (MOS) such as goserelin are equally efficacious to non-pharmacological ovarian suppression (NPOS) methods, which includes surgical oophorectomy and ovarian irradiation [15]. Furthermore, recent data from Suppression of Ovarian Function Trial (SOFT) and Tamoxifen and Exemastane Trial (TEXT) indicated the superiority of combining AI with goserelin in the cohort of adjuvant premenopausal patients younger than 35 years, may potentially result in an increase utilization of goserelin in the future [29]. In this study, we analyzed RNA-seq datasets from the ICGC database of very young breast cancer patients. We defined the risk prediction model of OS using 5 target genes, and 7 genes use for RFS model. There are two common genes (ASAP1 and NDST1) to build of risk prediction models for OS and RFS, and he high-risk group had significantly worse prognosis compared to low-risk group in both models. The results of ROC curve showed that the model of very young breast cancer patients we constructed have better classifier performance compared to all patients' model of breast cancer. Thus, the above results demonstrated that the risk prediction models constructed with goserelin target genes can well distinguish the prognosis of patients, especially for very young breast cancer patients.

Although a combination of multiple genes manifests more efficiently than a single gene as a diagnostic or prognostic biomarker, the predictive role of single gene was also explored in our study. CORO1A is highly expressed in cells of the haematopoietic lineage, where it has been predominantly investigated in lymphocytes, macrophages, mast cells and neutrophils [30], but has not been reported in tumors. Annexin A5 (ANXA5) is a member of the calcium and phospholipid binding protein family called the annexins, which bind phosphatidylserine (PS) with high affinity. Due to its preferential PS binding property, AnxA5 has been utilized as a marker for the detection of cells undergoing apoptosis [31]. Our data indicated that the expression of CORO1A and ANXA5 are significantly associated with multiple clinic-pathological features of breast cancer, such as age, menopause status, disease stage, nodal metastasis status, and molecular subtypes. Dipeptidyl peptidase (DPP)4 is a membrane-bound protein found in many cell types of the body, and a soluble form is present in body fluids [32]. Nazarian et al. found that DPP4 was reduced in mice with progressive invasive prostate cancer, and DPP4 activity could be used alone or in combination, the latter being more likely, with other markers of prostate cancer as an indicator of metastatic disease [33]. DPP4 as the composed gene of risk prediction models in prostate cancer, its expression is significantly related to multiple clinic-pathological factors and survival in our results. These findings emphasize the important role of the target genes of goserelin in constructing tumor risk models and evaluating prognosis.

## Conclusion

In conclusion, this study presents a novel signature with demonstrated prognostic value similar in magnitude to that of clinical staging of breast and prostate cancer, and having added value in very young breast cancer patients. This signature can facilitate identification of new biomarkers which sensitive to goserelin, increase the using accuracy of goserelin and clarify the classification of disease molecular subtypes in breast and prostate cancer. Future experimental and clinical studies are necessary to produce a solid confirmation of our results.

## Supplementary Information

**Additional file 1: Figure S1.** The compound and compound target genes of goserelin. (a) Chemical structures of goserelin. (b) Protein–protein interaction network of target genes of goserelin. (c) 10 hub genes in protein–protein interaction network. **Figure S2.** Construction and evaluation of the very young breast cancer risk prediction model in ICGC. (a) Overall survival (OS) in univariate Cox regression of target genes. (b) Recurrence-free survival (RFS) in univariate Cox regression of target genes. (c) Lasso regression for genes in univariate Cox regression of OS. (d) Lasso regression for genes in univariate Cox regression of RFS. (e) Kaplan–Meier survival curve (OS and RFS) for patients with high-risk group and low-risk group. (f) ROC curve showed the predictive efficiency (OS and RFS) of the risk score. **Figure S3.** The differentially expression of representative target genes between male and female in multiple normal human tissues by GTEx data, *P < 0.05, **P < 0.01 and ***P < 0.001. **Table S1.** GO analysis of target genes of goserelin. **Table S2.** KEGG pathways enrichment analysis of target genes of goserelin.

## Data Availability

The dataset supporting the conclusions of this article is included within the article.

## Abbreviations

TCGA: The Cancer Genome Atlas (TCGA)
ICGC: International Cancer Genome Consortium
GTEx: The Genotype-Tissue Expression
GnRH: Gonadotrophin-releasing hormone
NP: Network pharmacology
DEGs: Differentially expressed genes
FDR: False discovery rate
PPI: Protein protein interaction
GO: Gene ontology
KEGG: Kyoto Encyclopedia of Genes and Genomes
BP: Biological process
CC: Cellular component
MF: Molecular function
RFS: Recurrence-free survival
OS: Overall survival
tdROC: Time-dependent receiver operating characteristic
CDF: Cumulative distribution function
TNBC: Triple negative breast cancer
MOS: Medical ovarian suppression
NPOS: Non-pharmacological ovarian suppression
SOFT: Suppression of Ovarian Function Trial
TEXT: Tamoxifen and Exemastane Trial
PS: Phosphatidylserine
