# Supplementary material for: Identification of predictors based on drug targets highlights accurate treatment of goserelin in breast and prostate cancer
Source: Cell Biosci. 2021 Jan 6;11:5. doi: 10.1186/s13578-020-00517-w (PMC7788753; doi:10.1186/s13578-020-00517-w)
Supplement: Supplementary file 1 — Additional file 1: Figure S1. The compound and compound target genes of goserelin. (a) Chemical structures of goserelin. (b) Protein–protein interaction network of target genes of goserelin. (c) 10 hub genes in protein–protein interaction network. Figure S2. Construction and evaluation of the very young breast cancer risk prediction model in ICGC. (a) Overall survival (OS) in univariate Cox regression of target genes. (b) Recurrence-free survival (RFS) in univariate Cox regression of target genes. (c) Lasso regression for genes in univariate Cox regression of OS. (d) Lasso regression for genes in univariate Cox regression of RFS. (e) Kaplan–Meier survival curve (OS and RFS) for patients with high-risk group and low-risk group. (f) ROC curve showed the predictive efficiency (OS and RFS) of the risk score. Figure S3. The differentially expression of representative target genes between male and female in multiple normal human tissues by GTEx data, *P < 0.05, **P < 0.01 and ***P < 0.001. Table S1. GO analysis of target genes of goserelin. Table S2. KEGG pathways enrichment analysis of target genes of goserelin. [file 13578_2020_517_MOESM1_ESM.doc]

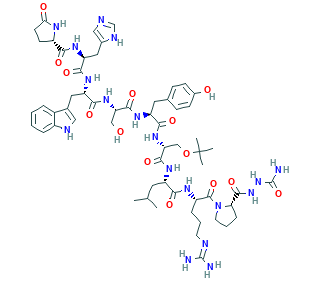


a

**
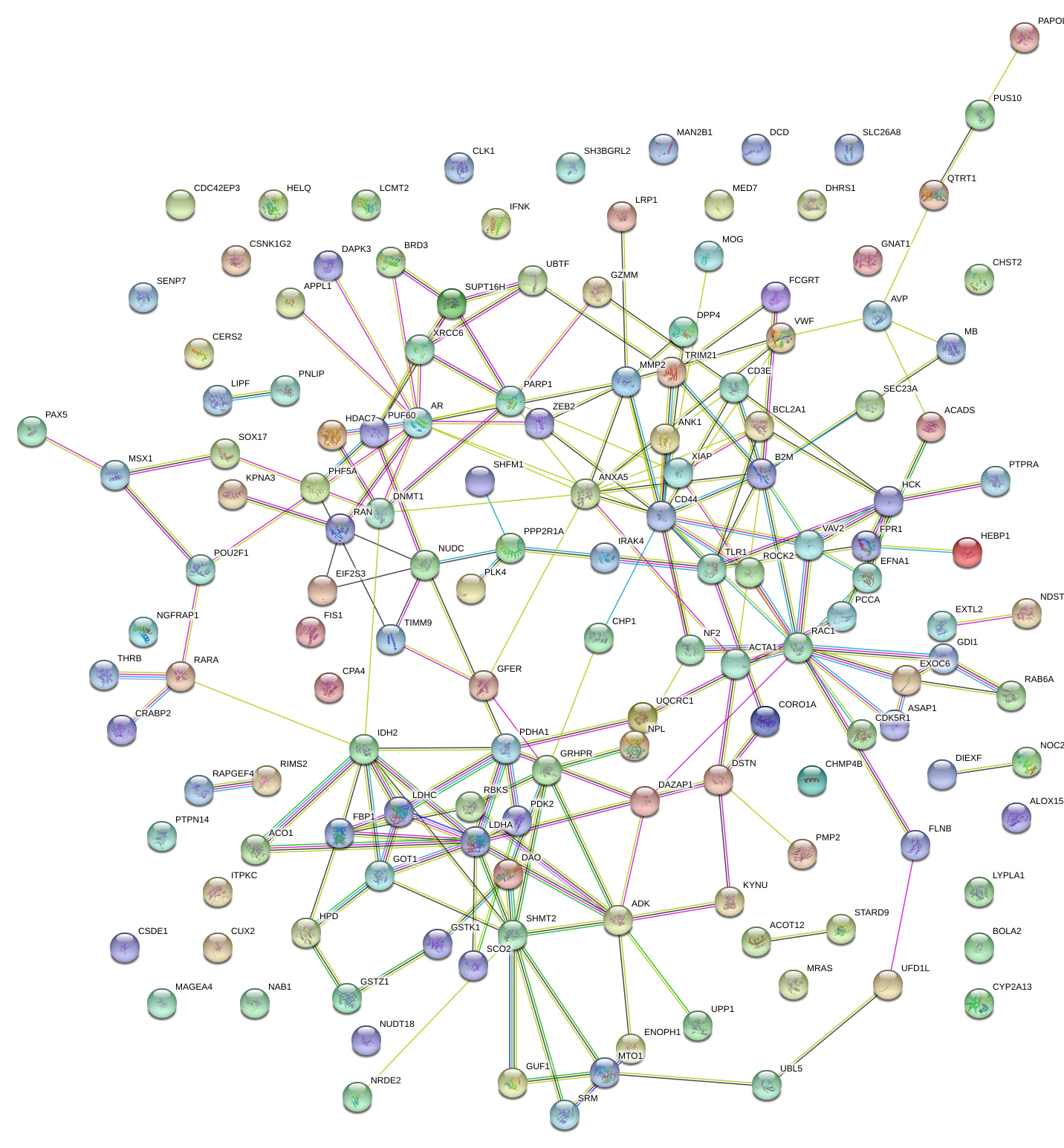
**

b


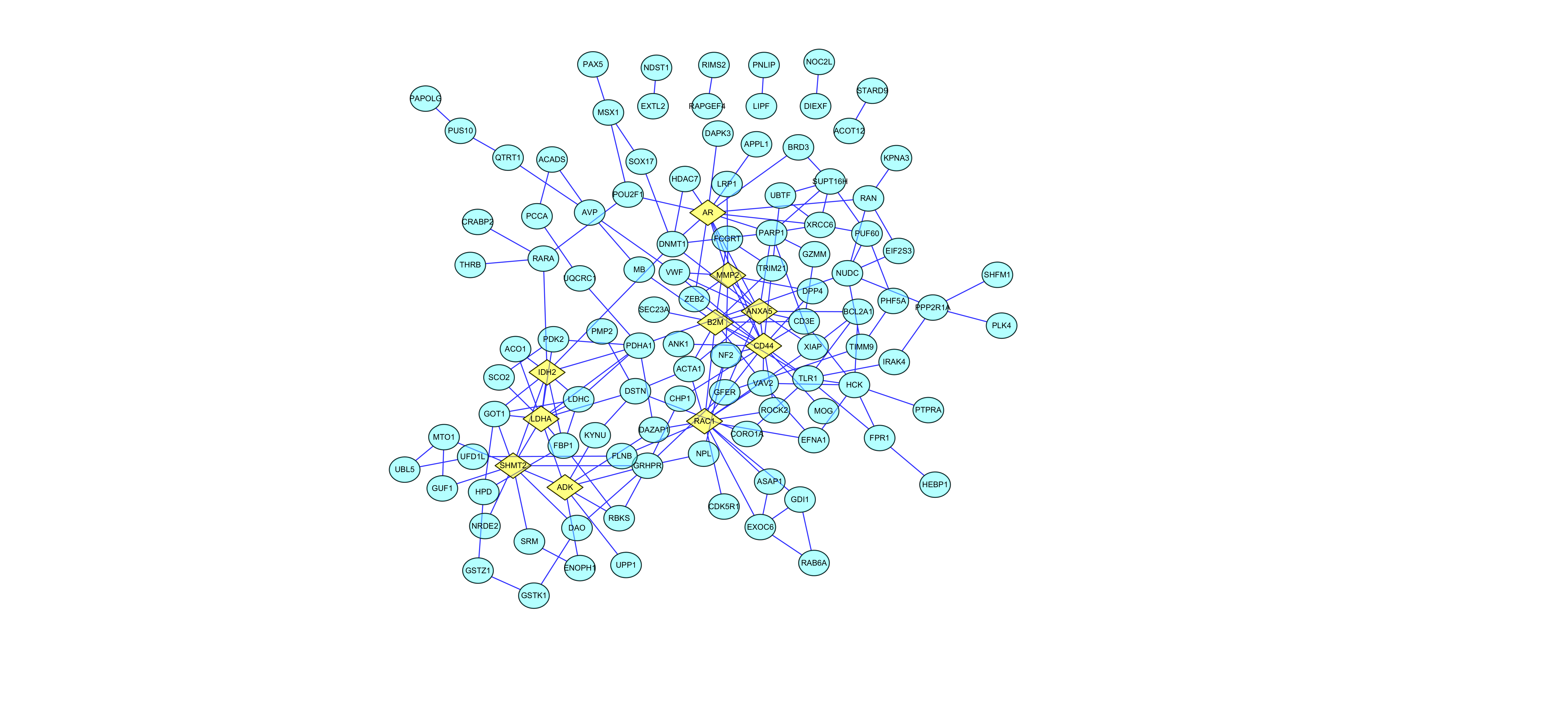
c

**Figure S1:** The compound and compound target genes of goserelin. (a) Chemical structures of goserelin. (b) Protein-protein interaction network of target genes of goserelin. (c) 10 hub genes in protein-protein interaction network.

a b


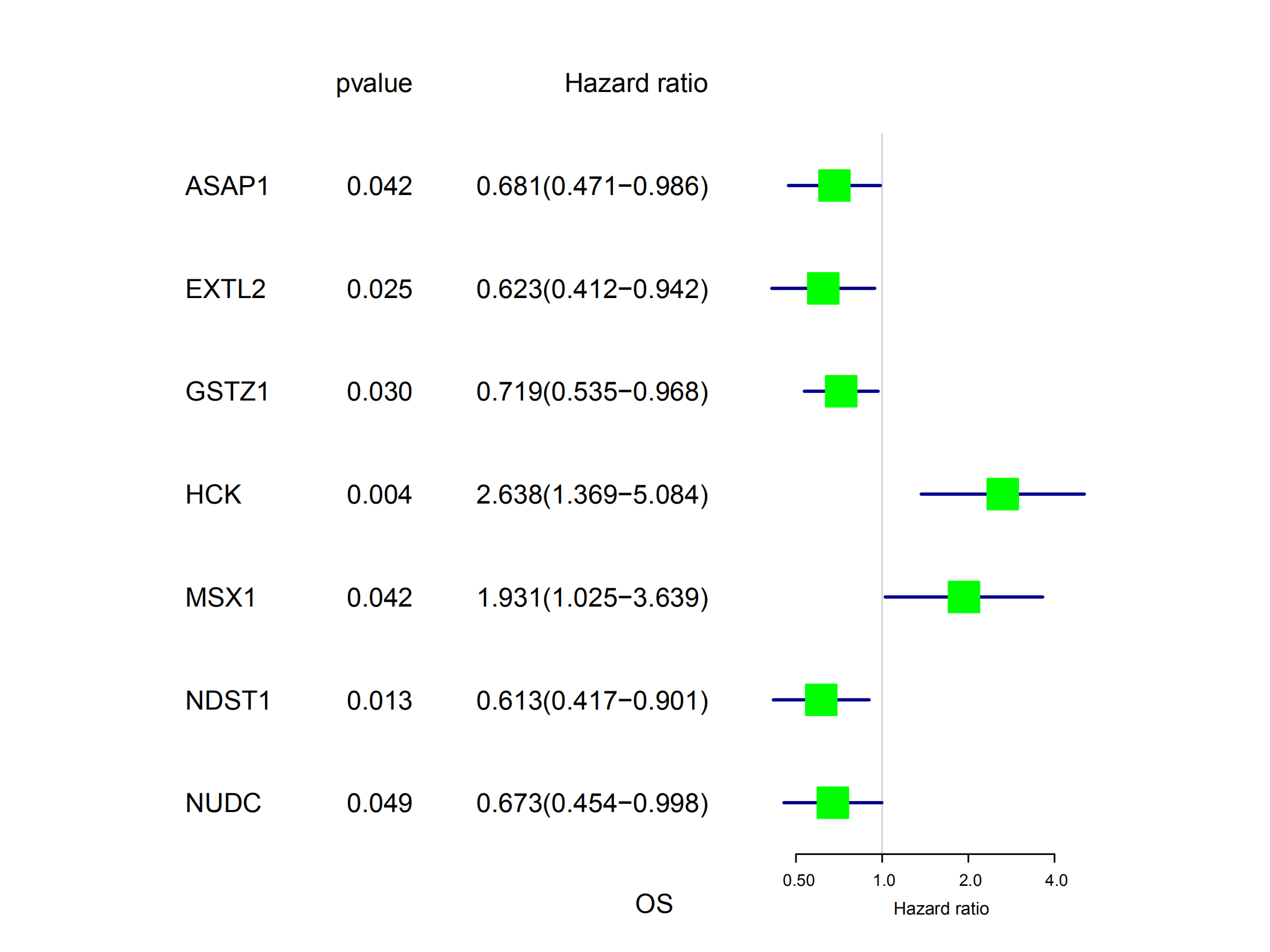

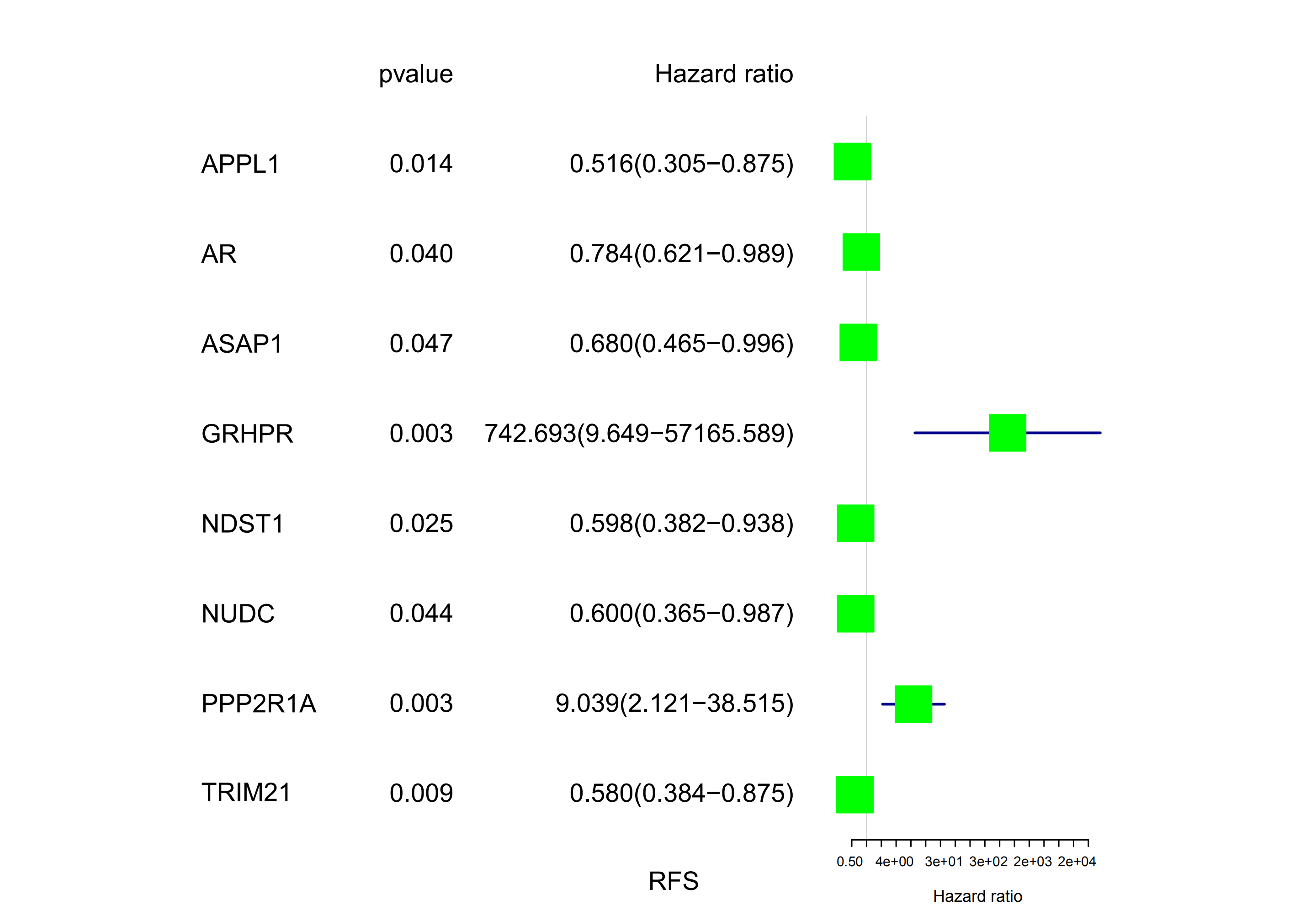


c


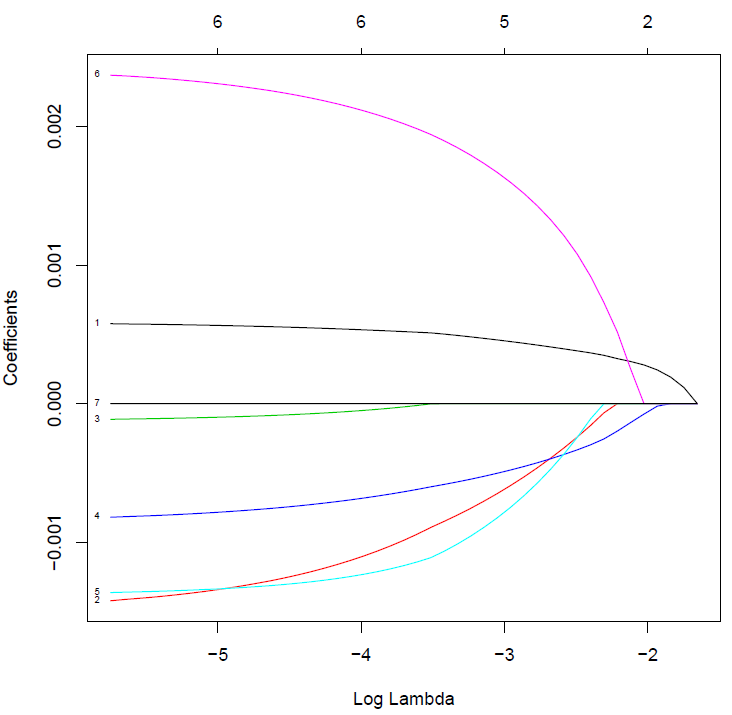

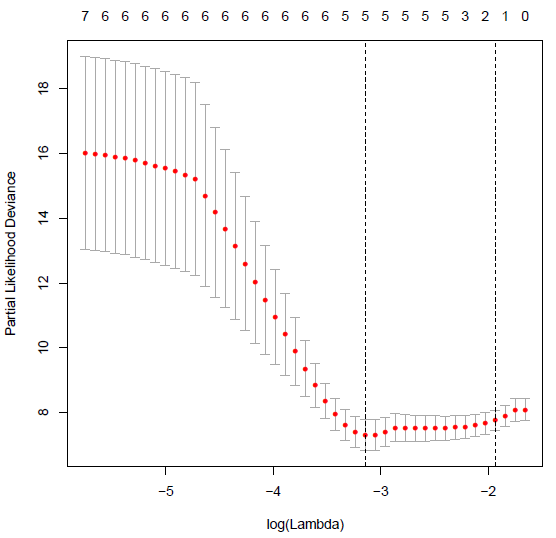


| **Gene** | **Coef** |
| --- | --- |
| HCK | 0.000471125 |
| ASAP1 | -0.000703053 |
| NDST1 | -0.000523865 |
| EXTL2 | -0.000891968 |
| MSX1 | 0.001732962 |

d


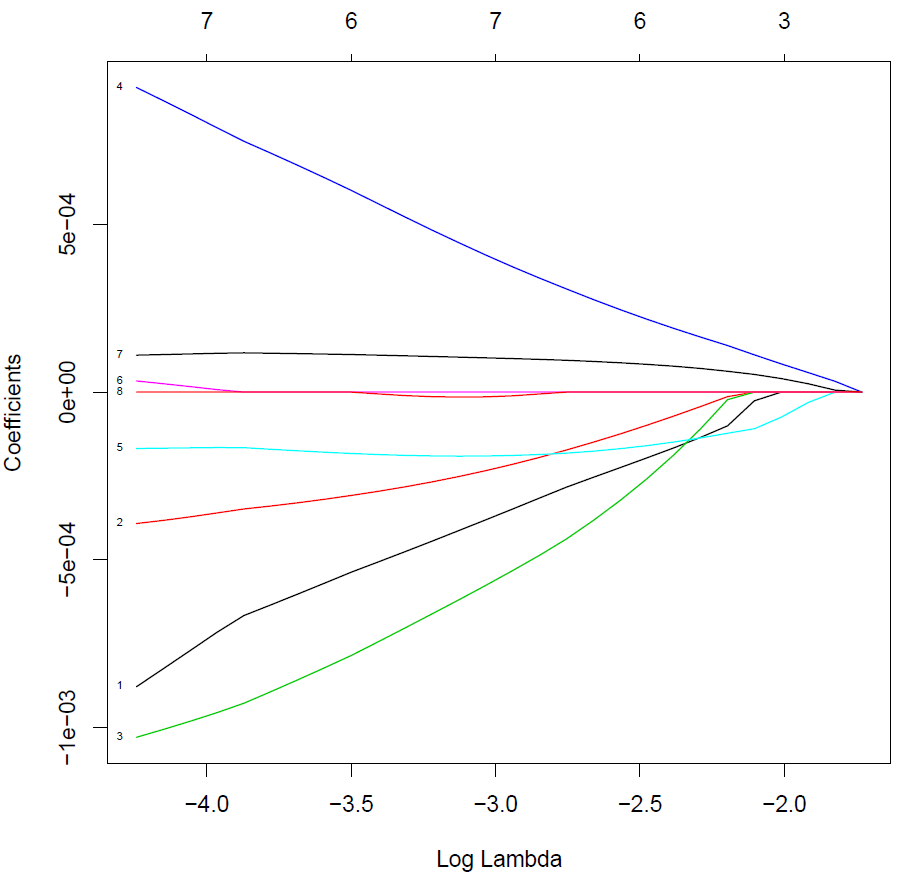

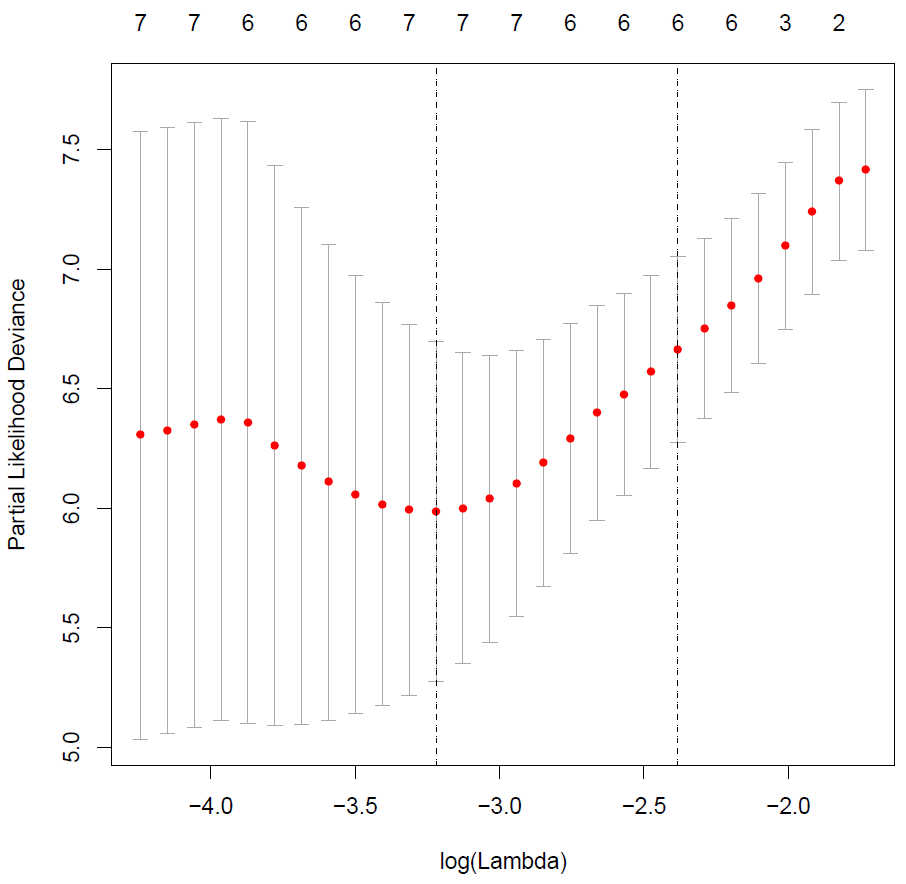


| **Gene** | **Coef** |
| --- | --- |
| APPL1 | -0.000444584 |
| AR | -0.000267821 |
| ASAP1 | -0.0006615208 |
| GRHPR | 0.00048319266 |
| NDST1 | -0.0001903003 |
| PPP2R1A | 0.00010628073 |
| TRIM21 | -1.363479e-05 |

e


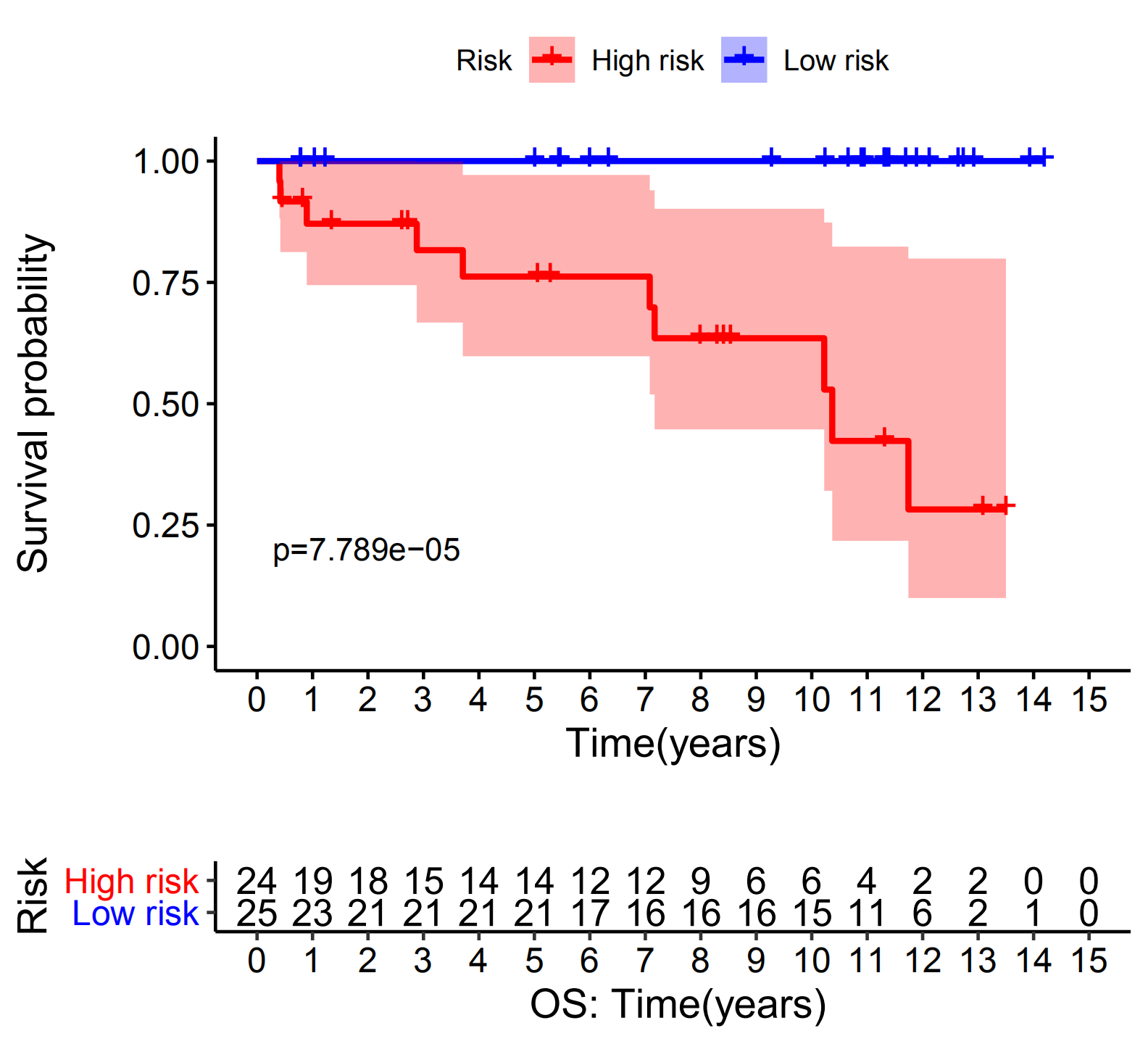

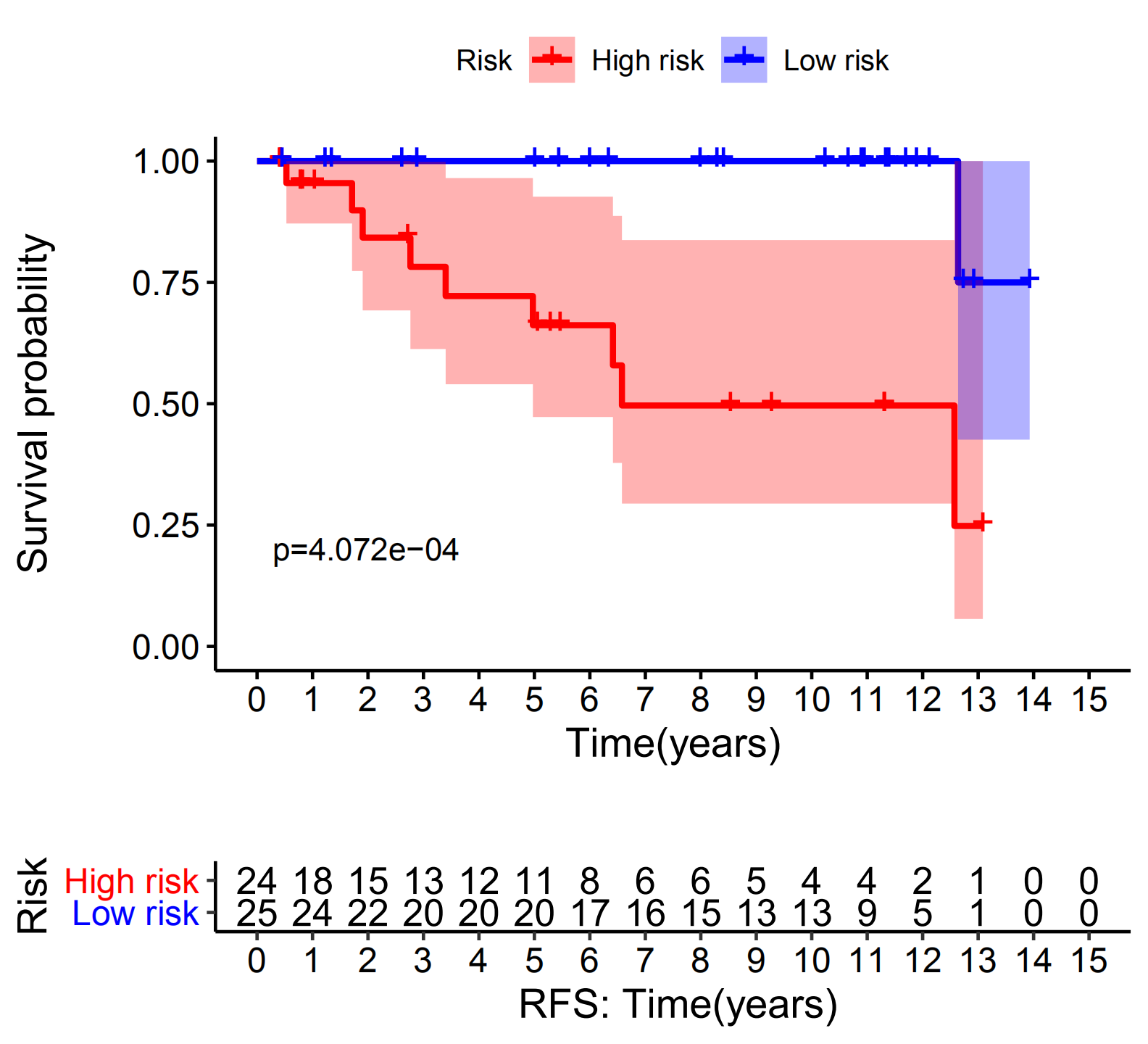


f


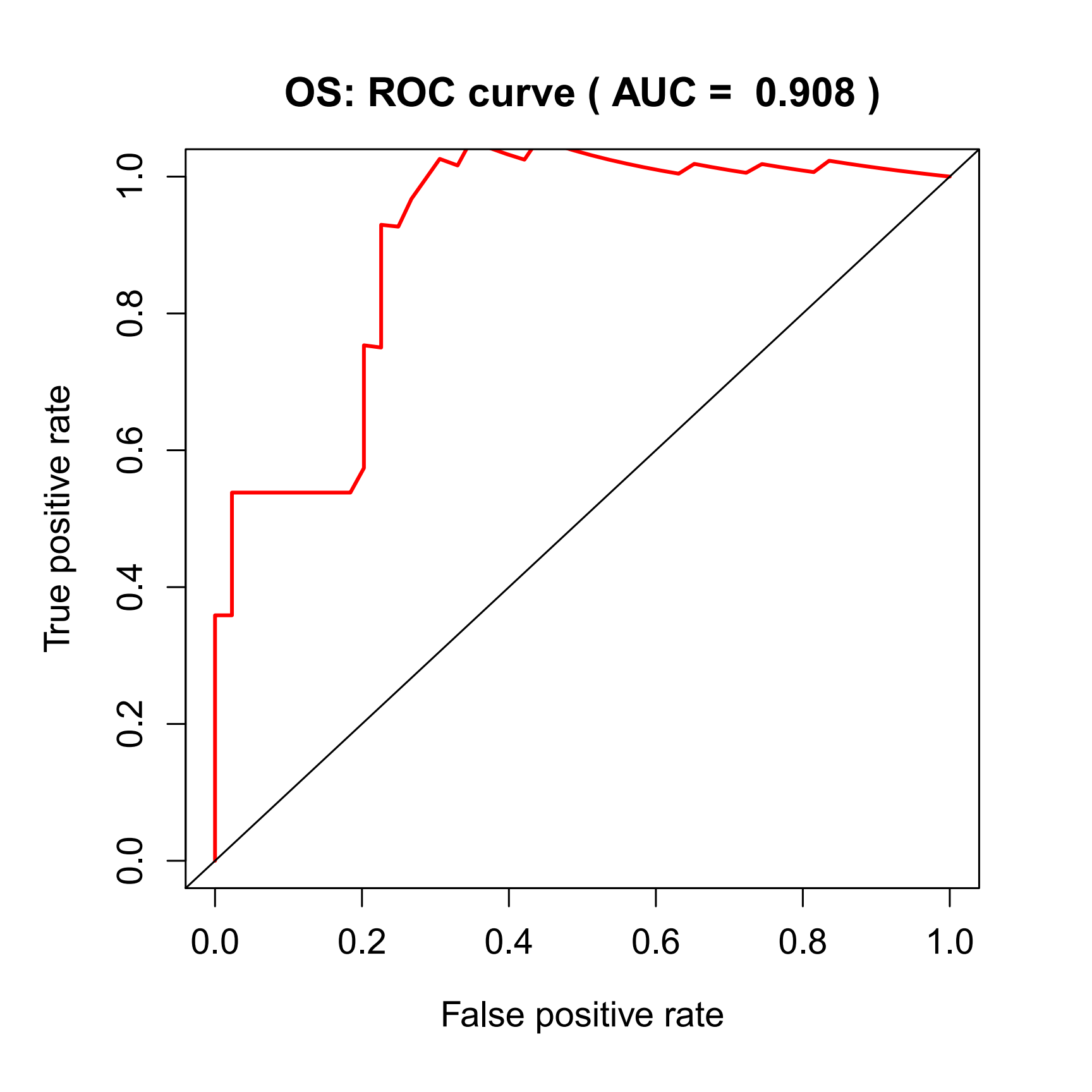

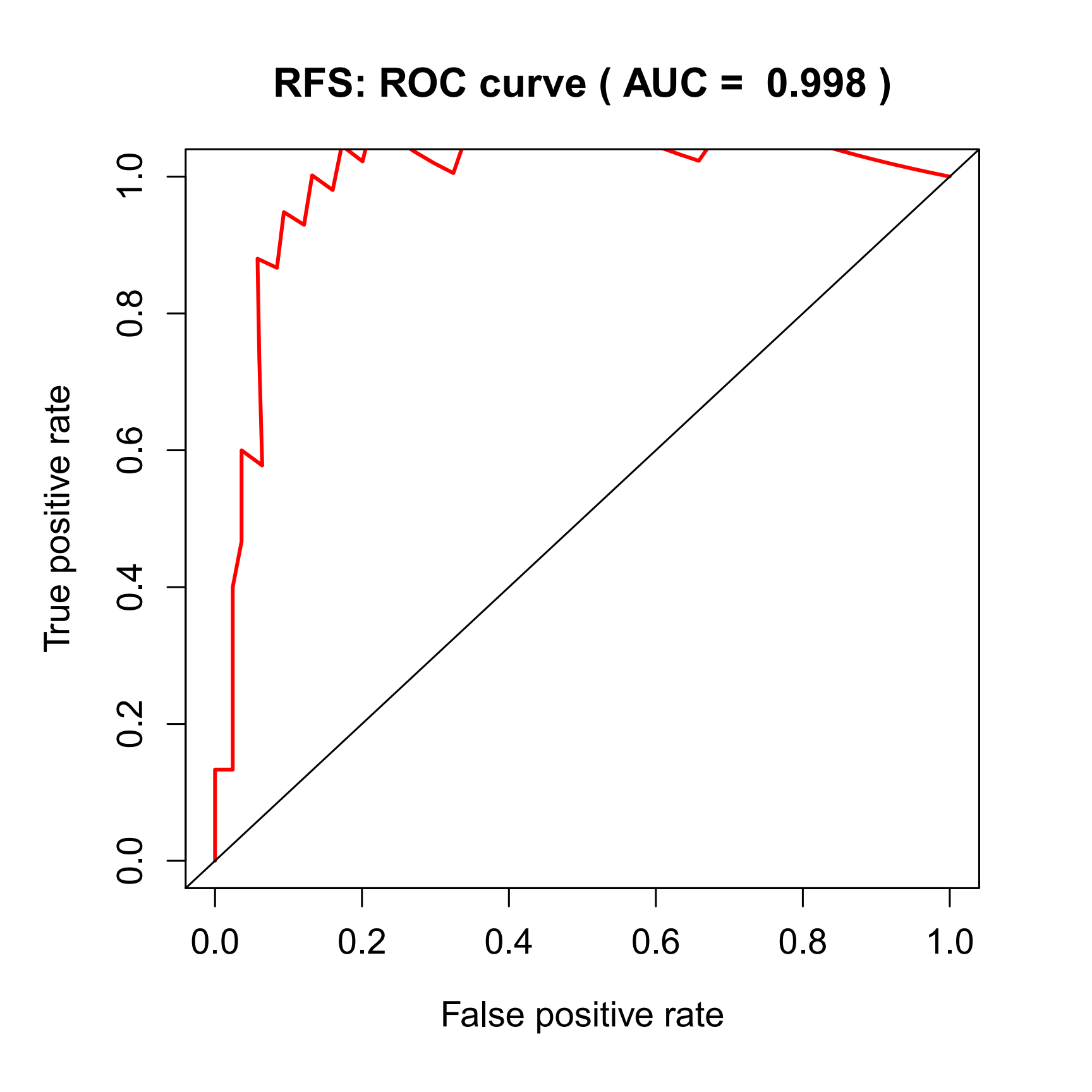


**Figure S2:** Construction and evaluation of the very young breast cancer risk prediction model in ICGC. (a) Overall survival (OS) in univariate Cox regression of target genes. (b) Recurrence-free survival (RFS) in univariate Cox regression of target genes. (c) Lasso regression for genes in univariate Cox regression of OS. (d) Lasso regression for genes in univariate Cox regression of RFS. (e) Kaplan-Meier survival curve (OS and RFS) for patients with high-risk group and low-risk group. (f) ROC curve showed the predictive efficiency (OS and RFS) of the risk score.


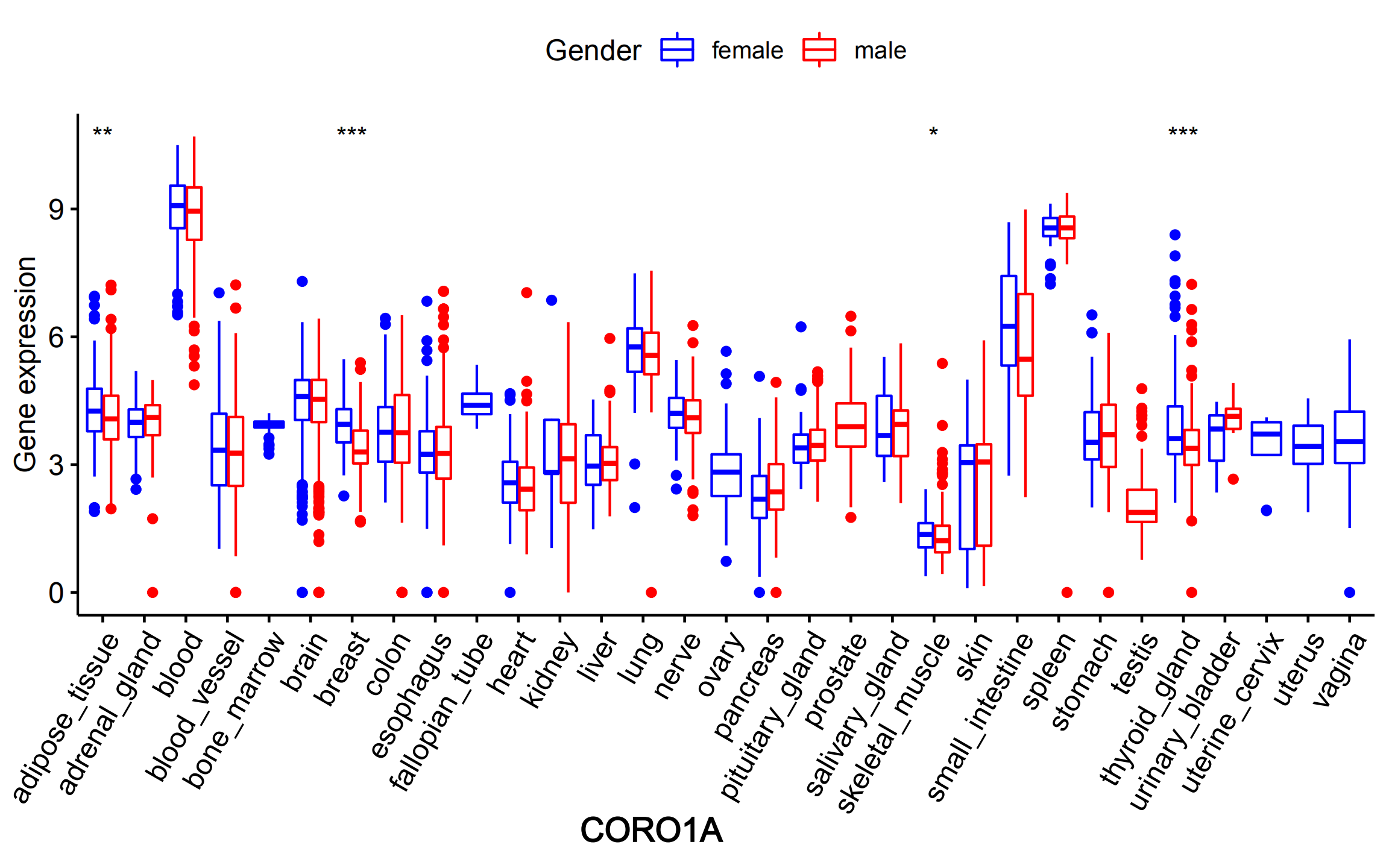

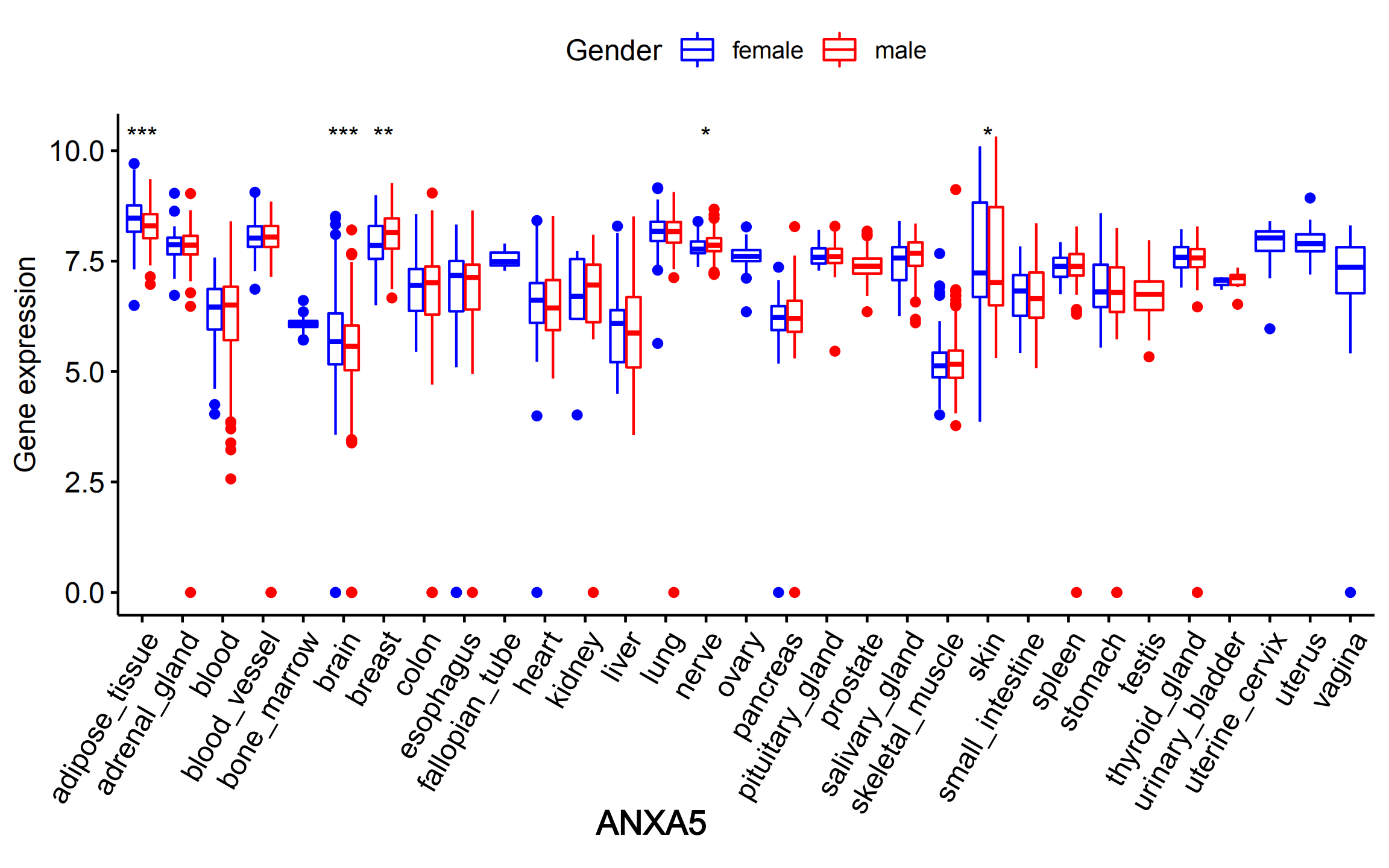


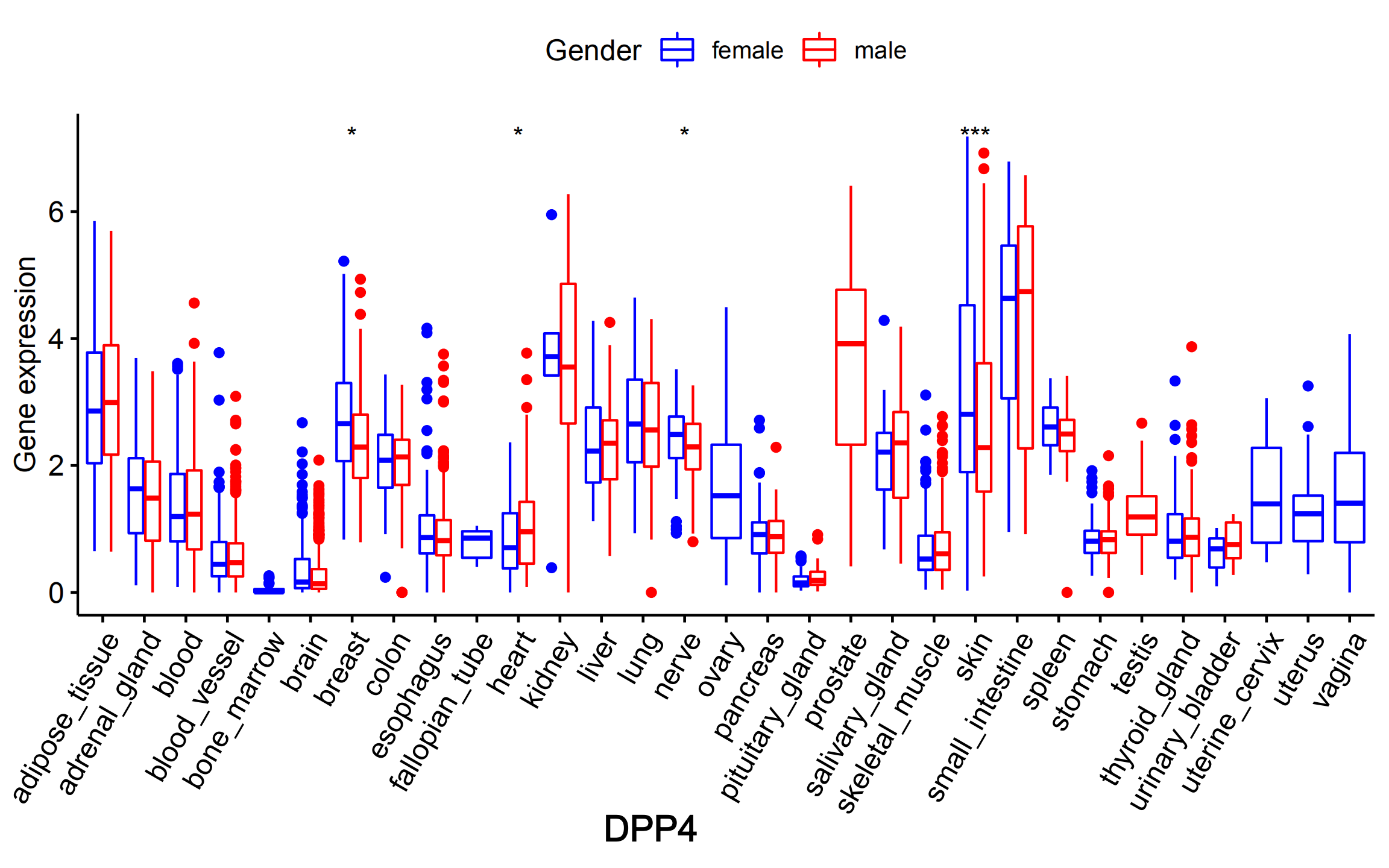


**Figure S3:** The differentially expression of representative target genes between male and female in multiple normal human tissues by GTEx data, **P* < 0.05, ***P* < 0.01 and ****P* < 0.001.

Table S1: GO analysis of target genes of goserelin

| **GO** | **Description** | **Gene** | **P adjust** |
| --- | --- | --- | --- |
| BP | ephrin receptor signaling pathway | MMP2/VAV2/RAC1/ROCK2/EFNA1/CDK5R1 | 0.003071295 |
| BP | nucleotide biosynthetic process | FBP1/IDH2/ADK/KYNU/PDHA1/GSTZ1/LDHC/LDHA/PARP1/PDK2 | 0.003071295 |
| BP | nucleoside phosphate biosynthetic process | FBP1/IDH2/ADK/KYNU/PDHA1/GSTZ1/LDHC/LDHA/PARP1/PDK2 | 0.003071295 |
| BP | organic acid catabolic process | HPD/KYNU/ACADS/GSTZ1/DAO/GOT1/CHP1/PCCA | 0.005840635 |
| BP | carboxylic acid catabolic process | HPD/KYNU/ACADS/GSTZ1/DAO/GOT1/CHP1/PCCA | 0.005840635 |
| BP | acetyl-CoA metabolic process | KYNU/PDHA1/GSTZ1/PDK2 | 0.005840635 |
| BP | purine ribonucleotide biosynthetic process | FBP1/ADK/PDHA1/GSTZ1/LDHC/LDHA/PARP1/PDK2 | 0.005840635 |
| BP | ribonucleotide biosynthetic process | FBP1/ADK/PDHA1/GSTZ1/LDHC/LDHA/PARP1/PDK2 | 0.005840635 |
| BP | regulation of cell morphogenesis | CORO1A/GDI1/CRABP2/RAC1/ZEB2/HCK/DAPK3/EFNA1/CDK5R1/RIMS2 | 0.005840635 |
| BP | purine nucleotide biosynthetic process | FBP1/ADK/PDHA1/GSTZ1/LDHC/LDHA/PARP1/PDK2 | 0.005840635 |
| BP | ribose phosphate biosynthetic process | FBP1/ADK/PDHA1/GSTZ1/LDHC/LDHA/PARP1/PDK2 | 0.005840635 |
| BP | acetyl-CoA biosynthetic process from pyruvate | PDHA1/GSTZ1/PDK2 | 0.005840635 |
| BP | coenzyme metabolic process | FBP1/IDH2/ENOPH1/KYNU/PDHA1/GSTZ1/LDHA/PDK2/PCCA | 0.005840635 |
| BP | positive regulation of cytoskeleton organization | CORO1A/RAC1/ROCK2/HCK/NF2/CDK5R1/PLK4 | 0.005840635 |
| BP | pyruvate metabolic process | FBP1/PDHA1/GSTZ1/LDHC/LDHA/PDK2 | 0.005840635 |
| BP | purine-containing compound biosynthetic process | FBP1/ADK/PDHA1/GSTZ1/LDHC/LDHA/PARP1/PDK2 | 0.005840635 |
| BP | dicarboxylic acid metabolic process | IDH2/KYNU/GOT1/GRHPR/LIPF | 0.007135354 |
| BP | signal transduction involved in regulation of gene expression | PARP1/SOX17/MSX1 | 0.008847946 |
| BP | alpha-amino acid catabolic process | HPD/KYNU/GSTZ1/DAO/GOT1 | 0.008847946 |
| BP | coenzyme biosynthetic process | FBP1/IDH2/KYNU/PDHA1/GSTZ1/LDHA/PDK2 | 0.010059576 |
| BP | acetyl-CoA biosynthetic process | PDHA1/GSTZ1/PDK2 | 0.010059576 |
| BP | cell-substrate adhesion | CORO1A/CD3E/RAC1/ROCK2/NF2/DAPK3/EFNA1/VWF | 0.010059576 |
| BP | response to metal ion | FBP1/GDI1/B2M/PARP1/GOT1/ANXA5/ACTA1/ACO1 | 0.011665832 |
| BP | regulation of cell-matrix adhesion | CD3E/RAC1/ROCK2/NF2/DAPK3 | 0.011985351 |
| BP | sulfur compound biosynthetic process | GSTK1/ENOPH1/PDHA1/GSTZ1/NDST1/PDK2 | 0.011985351 |
| BP | sulfur compound metabolic process | GSTK1/ENOPH1/KYNU/PDHA1/GSTZ1/NDST1/PDK2/PCCA | 0.011985351 |
| BP | cell growth | FBP1/PPP2R1A/GDI1/CRABP2/ZEB2/SOX17/CDK5R1/MSX1/RIMS2 | 0.012883986 |
| BP | cellular amino acid catabolic process | HPD/KYNU/GSTZ1/DAO/GOT1 | 0.012883986 |
| BP | response to interferon-gamma | TRIM21/B2M/KYNU/HCK/FLNB/DAPK3 | 0.012883986 |
| BP | positive regulation of supramolecular fiber organization | CORO1A/RAC1/ROCK2/HCK/NF2/CDK5R1 | 0.014251151 |
| BP | negative regulation of neurogenesis | ASAP1/GDI1/B2M/THRB/IDH2/EFNA1/CDK5R1 | 0.014407152 |
| BP | aromatic amino acid family catabolic process | HPD/KYNU/GSTZ1 | 0.014407152 |
| BP | regulation of defense response to virus by virus | B2M/RAC1/HCK | 0.017212944 |
| BP | regulation of cell growth | FBP1/PPP2R1A/GDI1/CRABP2/SOX17/CDK5R1/MSX1/RIMS2 | 0.019074928 |
| BP | alpha-amino acid metabolic process | HPD/ENOPH1/KYNU/GSTZ1/DAO/GOT1 | 0.019074928 |
| BP | negative regulation of nervous system development | ASAP1/GDI1/B2M/THRB/IDH2/EFNA1/CDK5R1 | 0.019074928 |
| BP | negative regulation of neuron differentiation | ASAP1/GDI1/B2M/THRB/EFNA1/CDK5R1 | 0.019584362 |
| BP | hippocampus development | RARA/ZEB2/NF2/CDK5R1 | 0.020453033 |
| BP | tricarboxylic acid cycle | IDH2/PDHA1/ACO1 | 0.021116191 |
| BP | cofactor biosynthetic process | FBP1/IDH2/KYNU/PDHA1/GSTZ1/LDHA/PDK2 | 0.021116191 |
| BP | citrate metabolic process | IDH2/PDHA1/ACO1 | 0.0217533 |
| BP | hormone-mediated signaling pathway | RARA/THRB/PARP1/AR/APPL1/RAN | 0.0217533 |
| BP | cellular carbohydrate biosynthetic process | FBP1/EXTL2/NDST1/GOT1 | 0.022061713 |
| BP | regulation of protein targeting to membrane | GDI1/CDK5R1/CHP1 | 0.022061713 |
| BP | negative regulation of neuron projection development | ASAP1/GDI1/B2M/EFNA1/CDK5R1 | 0.022061713 |
| BP | aerobic respiration | UQCRC1/IDH2/PDHA1/ACO1 | 0.022192502 |
| BP | small molecule catabolic process | HPD/KYNU/ACADS/GSTZ1/DAO/GOT1/CHP1/PCCA | 0.022211595 |
| BP | purine nucleoside triphosphate metabolic process | FBP1/UQCRC1/ADK/LDHC/LDHA/PARP1/RAN | 0.023298758 |
| BP | regulation of actin cytoskeleton organization | CORO1A/RAC1/ROCK2/HCK/NF2/DAPK3/CDK5R1 | 0.023298758 |
| BP | negative regulation of cell development | ASAP1/GDI1/B2M/THRB/IDH2/EFNA1/CDK5R1 | 0.023298758 |
| BP | aromatic amino acid family metabolic process | HPD/KYNU/GSTZ1 | 0.023950417 |
| BP | tricarboxylic acid metabolic process | IDH2/PDHA1/ACO1 | 0.023950417 |
| BP | nucleoside triphosphate metabolic process | FBP1/UQCRC1/ADK/LDHC/LDHA/PARP1/RAN | 0.025110696 |
| BP | purine nucleoside triphosphate biosynthetic process | FBP1/ADK/LDHC/LDHA/PARP1 | 0.025110696 |
| BP | pallium development | PAX5/RARA/ZEB2/NF2/CDK5R1 | 0.025110696 |
| BP | protein localization to nucleus | PARP1/NF2/APPL1/RAN/MSX1/CHP1 | 0.025110696 |
| BP | tyrosine metabolic process | HPD/GSTZ1 | 0.025110696 |
| BP | regulation of melanin biosynthetic process | ZEB2/APPL1 | 0.025110696 |
| BP | phenol-containing compound biosynthetic process | ZEB2/DAO/APPL1 | 0.025110696 |
| BP | regulation of cellular component size | CORO1A/GDI1/VAV2/CRABP2/RAC1/HCK/CDK5R1 | 0.025110696 |
| BP | positive regulation of protein complex assembly | ASAP1/CORO1A/RAC1/PARP1/HCK/CDK5R1 | 0.025110696 |
| BP | regulation of cell size | GDI1/VAV2/CRABP2/RAC1/CDK5R1 | 0.025110696 |
| BP | organic hydroxy compound biosynthetic process | LDHC/ZEB2/DAO/APPL1/GOT1/RAN | 0.025110696 |
| BP | cellular response to interferon-gamma | TRIM21/B2M/HCK/FLNB/DAPK3 | 0.025110696 |
| BP | regulation of cell adhesion mediated by integrin | CD3E/EFNA1/DPP4 | 0.025110696 |
| BP | L-phenylalanine metabolic process | HPD/GSTZ1 | 0.025110696 |
| BP | L-phenylalanine catabolic process | HPD/GSTZ1 | 0.025110696 |
| BP | heparan sulfate proteoglycan biosynthetic process, polysaccharide chain biosynthetic process | EXTL2/NDST1 | 0.025110696 |
| BP | positive regulation of intracellular estrogen receptor signaling pathway | PARP1/AR | 0.025110696 |
| BP | regulation of cell-cell adhesion mediated by integrin | CD3E/DPP4 | 0.025110696 |
| BP | positive regulation of centrosome cycle | ROCK2/PLK4 | 0.025110696 |
| BP | negative regulation of histone H3-K9 methylation | DNMT1/PAX5 | 0.025110696 |
| BP | erythrose 4-phosphate/phosphoenolpyruvate family amino acid metabolic process | HPD/GSTZ1 | 0.025110696 |
| BP | erythrose 4-phosphate/phosphoenolpyruvate family amino acid catabolic process | HPD/GSTZ1 | 0.025110696 |
| BP | regulation of aspartic-type peptidase activity | ROCK2/EFNA1 | 0.025110696 |
| BP | positive regulation of extrinsic apoptotic signaling pathway in absence of ligand | PPP2R1A/DAPK3 | 0.025110696 |
| BP | acyl-CoA metabolic process | KYNU/PDHA1/GSTZ1/PDK2 | 0.025110696 |
| BP | thioester metabolic process | KYNU/PDHA1/GSTZ1/PDK2 | 0.025110696 |
| BP | purine nucleoside monophosphate biosynthetic process | FBP1/ADK/LDHC/LDHA/PARP1 | 0.025110696 |
| BP | purine ribonucleoside monophosphate biosynthetic process | FBP1/ADK/LDHC/LDHA/PARP1 | 0.025110696 |
| BP | regulation of axonogenesis | GDI1/CRABP2/ZEB2/EFNA1/CDK5R1 | 0.025110696 |
| BP | forebrain development | PAX5/RARA/B2M/ZEB2/NF2/CDK5R1/MSX1 | 0.025110696 |
| BP | steroid hormone mediated signaling pathway | RARA/THRB/PARP1/AR/RAN | 0.025110696 |
| BP | regulation of neuron projection development | ASAP1/GDI1/B2M/CRABP2/ZEB2/EFNA1/CDK5R1/RIMS2 | 0.025110696 |
| BP | response to steroid hormone | RARA/THRB/PARP1/AR/GOT1/RAN/ACTA1 | 0.025110696 |
| BP | negative regulation of cell growth | FBP1/PPP2R1A/SOX17/CDK5R1/MSX1 | 0.025110696 |
| BP | intracellular receptor signaling pathway | RARA/THRB/CRABP2/PARP1/AR/RAN | 0.025110696 |
| BP | face development | MMP2/RARA/MSX1 | 0.025110696 |
| BP | regulation of protein localization to membrane | GDI1/AR/APPL1/CDK5R1/CHP1 | 0.025110696 |
| BP | regulation of actin filament-based process | CORO1A/RAC1/ROCK2/HCK/NF2/DAPK3/CDK5R1 | 0.025110696 |
| BP | nucleoside triphosphate biosynthetic process | FBP1/ADK/LDHC/LDHA/PARP1 | 0.025110696 |
| BP | negative regulation of cell projection organization | ASAP1/GDI1/B2M/EFNA1/CDK5R1 | 0.025110696 |
| BP | limbic system development | RARA/ZEB2/NF2/CDK5R1 | 0.025110696 |
| BP | pyridine nucleotide metabolic process | FBP1/IDH2/KYNU/PDHA1/LDHA | 0.025110696 |
| BP | nicotinamide nucleotide metabolic process | FBP1/IDH2/KYNU/PDHA1/LDHA | 0.025110696 |
| BP | regulation of cell fate specification | AR/SOX17 | 0.025110696 |
| BP | regulation of secondary metabolite biosynthetic process | ZEB2/APPL1 | 0.025110696 |
| BP | regulation of protein stability | TRIM21/SOX17/NF2/EFNA1/MSX1/CHP1 | 0.025110696 |
| BP | cellular carbohydrate metabolic process | FBP1/IDH2/EXTL2/NDST1/PDK2/GOT1 | 0.025770539 |
| BP | positive regulation of stress fiber assembly | RAC1/ROCK2/NF2 | 0.027402781 |
| BP | pyridine-containing compound metabolic process | FBP1/IDH2/KYNU/PDHA1/LDHA | 0.027683626 |
| BP | negative regulation of tyrosine phosphorylation of STAT protein | PPP2R1A/NF2 | 0.028100678 |
| BP | negative regulation of dendritic spine development | ASAP1/EFNA1 | 0.028100678 |
| BP | ribonucleoside monophosphate biosynthetic process | FBP1/ADK/LDHC/LDHA/PARP1 | 0.028129925 |
| BP | regulation of protein localization to nucleus | PARP1/NF2/RAN/CHP1 | 0.028616277 |
| BP | regulation of purine nucleotide biosynthetic process | FBP1/GSTZ1/PARP1/PDK2 | 0.029260073 |
| BP | regulation of nucleotide biosynthetic process | FBP1/GSTZ1/PARP1/PDK2 | 0.029586165 |
| BP | thioester biosynthetic process | PDHA1/GSTZ1/PDK2 | 0.029586165 |
| BP | acyl-CoA biosynthetic process | PDHA1/GSTZ1/PDK2 | 0.029586165 |
| BP | positive regulation of T cell activation | CORO1A/RARA/CD3E/RAC1/DPP4 | 0.029586165 |
| BP | regulation of cofactor metabolic process | FBP1/RAC1/GSTZ1/PDK2 | 0.029586165 |
| BP | regulation of cell morphogenesis involved in differentiation | GDI1/CRABP2/RAC1/ZEB2/EFNA1/CDK5R1 | 0.029586165 |
| BP | regulation of secondary metabolic process | ZEB2/APPL1 | 0.029804158 |
| BP | T cell costimulation | CD3E/RAC1/DPP4 | 0.031533343 |
| BP | oxidoreduction coenzyme metabolic process | FBP1/IDH2/KYNU/PDHA1/LDHA | 0.031656299 |
| BP | nucleoside monophosphate biosynthetic process | FBP1/ADK/LDHC/LDHA/PARP1 | 0.032031159 |
| BP | lymphocyte costimulation | CD3E/RAC1/DPP4 | 0.032031159 |
| BP | regulation of sulfur metabolic process | GSTZ1/PDK2 | 0.032031159 |
| BP | dicarboxylic acid biosynthetic process | KYNU/GOT1 | 0.032031159 |
| BP | regulation of podosome assembly | ASAP1/HCK | 0.032031159 |
| BP | regulation of plasma membrane organization | ASAP1/AR | 0.032031159 |
| BP | carbohydrate biosynthetic process | FBP1/EXTL2/NDST1/PDK2/GOT1 | 0.034246439 |
| BP | regulation of cell-substrate adhesion | CD3E/RAC1/ROCK2/NF2/DAPK3 | 0.034246439 |
| BP | regulation of focal adhesion assembly | RAC1/ROCK2/DAPK3 | 0.034246439 |
| BP | regulation of cell-substrate junction assembly | RAC1/ROCK2/DAPK3 | 0.034246439 |
| BP | 2-oxoglutarate metabolic process | IDH2/GOT1 | 0.034246439 |
| BP | cell-cell adhesion mediated by integrin | CD3E/DPP4 | 0.034246439 |
| BP | positive regulation of amyloid-beta formation | ROCK2/EFNA1 | 0.034246439 |
| BP | negative regulation of ATP biosynthetic process | FBP1/PARP1 | 0.034246439 |
| BP | positive regulation of leukocyte cell-cell adhesion | CORO1A/RARA/CD3E/RAC1/DPP4 | 0.034847109 |
| BP | cellular response to peptide hormone stimulus | ROCK2/PARP1/PDK2/APPL1/GOT1/ANXA5 | 0.034847109 |
| BP | positive regulation of actin filament bundle assembly | RAC1/ROCK2/NF2 | 0.034847109 |
| BP | response to nutrient | RARA/CD3E/KYNU/LDHA/PDK2 | 0.03503303 |
| BP | lactate metabolic process | LDHC/LDHA | 0.036990016 |
| BP | positive regulation of intracellular steroid hormone receptor signaling pathway | PARP1/AR | 0.036990016 |
| BP | positive regulation of protein polymerization | CORO1A/RAC1/HCK/CDK5R1 | 0.037500047 |
| BP | cell-matrix adhesion | CD3E/RAC1/ROCK2/NF2/DAPK3 | 0.038252493 |
| BP | Ras protein signal transduction | DNMT1/FBP1/GDI1/RAB6A/VAV2/RAC1/ROCK2 | 0.038906416 |
| BP | negative regulation of nucleotide biosynthetic process | FBP1/PARP1 | 0.039527349 |
| BP | short-chain fatty acid metabolic process | ACADS/PCCA | 0.039527349 |
| BP | negative regulation of purine nucleotide biosynthetic process | FBP1/PARP1 | 0.039527349 |
| BP | purine ribonucleoside triphosphate metabolic process | FBP1/UQCRC1/LDHC/LDHA/PARP1/RAN | 0.039527349 |
| BP | immune response-regulating cell surface receptor signaling pathway involved in phagocytosis | VAV2/RAC1/HCK/APPL1 | 0.039527349 |
| BP | Fc-gamma receptor signaling pathway involved in phagocytosis | VAV2/RAC1/HCK/APPL1 | 0.039527349 |
| BP | nucleoside bisphosphate metabolic process | KYNU/PDHA1/GSTZ1/PDK2 | 0.039527349 |
| BP | ribonucleoside bisphosphate metabolic process | KYNU/PDHA1/GSTZ1/PDK2 | 0.039527349 |
| BP | purine nucleoside bisphosphate metabolic process | KYNU/PDHA1/GSTZ1/PDK2 | 0.039527349 |
| BP | regulation of adherens junction organization | RAC1/ROCK2/DAPK3 | 0.039527349 |
| BP | purine ribonucleoside monophosphate metabolic process | FBP1/UQCRC1/ADK/LDHC/LDHA/PARP1 | 0.039527349 |
| BP | purine nucleoside monophosphate metabolic process | FBP1/UQCRC1/ADK/LDHC/LDHA/PARP1 | 0.039527349 |
| BP | ribonucleoside triphosphate metabolic process | FBP1/UQCRC1/LDHC/LDHA/PARP1/RAN | 0.039527349 |
| BP | developmental cell growth | GDI1/CRABP2/ZEB2/CDK5R1/RIMS2 | 0.039527349 |
| BP | podosome assembly | ASAP1/HCK | 0.039527349 |
| BP | cell adhesion mediated by integrin | CD3E/EFNA1/DPP4 | 0.039527349 |
| BP | nucleoside bisphosphate biosynthetic process | PDHA1/GSTZ1/PDK2 | 0.039527349 |
| BP | ribonucleoside bisphosphate biosynthetic process | PDHA1/GSTZ1/PDK2 | 0.039527349 |
| BP | purine nucleoside bisphosphate biosynthetic process | PDHA1/GSTZ1/PDK2 | 0.039527349 |
| BP | Fc-gamma receptor signaling pathway | VAV2/RAC1/HCK/APPL1 | 0.039527349 |
| BP | regulation of purine nucleotide metabolic process | FBP1/GSTZ1/PARP1/PDK2 | 0.039527349 |
| BP | developmental growth involved in morphogenesis | GDI1/CRABP2/ZEB2/CDK5R1/RIMS2 | 0.039527349 |
| BP | carboxylic acid biosynthetic process | FBP1/ENOPH1/KYNU/CRABP2/LDHC/LDHA/GOT1 | 0.039597008 |
| BP | organic acid biosynthetic process | FBP1/ENOPH1/KYNU/CRABP2/LDHC/LDHA/GOT1 | 0.039828218 |
| BP | positive regulation of cytokine production | XRCC6/RARA/B2M/CD3E/TLR1/ROCK2/APPL1 | 0.040060983 |
| BP | Fc receptor mediated stimulatory signaling pathway | VAV2/RAC1/HCK/APPL1 | 0.041371309 |
| BP | negative regulation of histone methylation | DNMT1/PAX5 | 0.041645342 |
| BP | positive regulation of amyloid precursor protein catabolic process | ROCK2/EFNA1 | 0.041645342 |
| BP | regulation of nucleotide metabolic process | FBP1/GSTZ1/PARP1/PDK2 | 0.041645342 |
| BP | nicotinamide nucleotide biosynthetic process | FBP1/IDH2/KYNU/LDHA | 0.042174789 |
| BP | pyridine nucleotide biosynthetic process | FBP1/IDH2/KYNU/LDHA | 0.042174789 |
| BP | regulation of supramolecular fiber organization | CORO1A/RAC1/ROCK2/HCK/NF2/CDK5R1 | 0.042681812 |
| BP | signal transduction in absence of ligand | PPP2R1A/APPL1/DAPK3 | 0.042921628 |
| BP | extrinsic apoptotic signaling pathway in absence of ligand | PPP2R1A/APPL1/DAPK3 | 0.042921628 |
| BP | ribonucleoside monophosphate metabolic process | FBP1/UQCRC1/ADK/LDHC/LDHA/PARP1 | 0.043126832 |
| BP | melanin biosynthetic process | ZEB2/APPL1 | 0.043370512 |
| BP | regulation of mitochondrial depolarization | PARP1/GOT1 | 0.043370512 |
| BP | pyridine-containing compound biosynthetic process | FBP1/IDH2/KYNU/LDHA | 0.043370512 |
| BP | purine ribonucleoside metabolic process | ENOPH1/ADK/RAN | 0.043370512 |
| BP | antibiotic metabolic process | IDH2/PDHA1/RAC1/ACO1 | 0.044089801 |
| BP | regulation of dendritic spine development | ASAP1/EFNA1/CDK5R1 | 0.044568368 |
| BP | negative regulation of growth | FBP1/PPP2R1A/SOX17/CDK5R1/MSX1 | 0.044786331 |
| BP | regulation of cellular amide metabolic process | RARA/GSTZ1/ROCK2/PDK2/DAPK3/EFNA1/ACO1 | 0.044786331 |
| BP | peptide hormone secretion | RAC1/RAPGEF4/ANXA5/RIMS2/DPP4 | 0.044786331 |
| BP | cellular response to steroid hormone stimulus | RARA/THRB/PARP1/AR/RAN | 0.044786331 |
| BP | melanin metabolic process | ZEB2/APPL1 | 0.044786331 |
| BP | glutathione derivative metabolic process | GSTK1/GSTZ1 | 0.044786331 |
| BP | glutathione derivative biosynthetic process | GSTK1/GSTZ1 | 0.044786331 |
| BP | telencephalon development | PAX5/RARA/ZEB2/NF2/CDK5R1 | 0.044786331 |
| BP | positive regulation of cell morphogenesis involved in differentiation | GDI1/CRABP2/RAC1/ZEB2 | 0.044786331 |
| BP | purine nucleoside metabolic process | ENOPH1/ADK/RAN | 0.045510881 |
| BP | regulation of animal organ morphogenesis | THRB/RAC1/AR/SOX17/MSX1 | 0.045641722 |
| BP | cellular amino acid metabolic process | HPD/ENOPH1/KYNU/GSTZ1/DAO/GOT1 | 0.045987263 |
| BP | positive regulation of cell-cell adhesion | CORO1A/RARA/CD3E/RAC1/DPP4 | 0.04641665 |
| BP | phagocytosis | CORO1A/RARA/VAV2/RAC1/HCK/APPL1 | 0.04641665 |
| BP | aspartate family amino acid biosynthetic process | ENOPH1/GOT1 | 0.04641665 |
| BP | positive regulation of interleukin-4 production | RARA/CD3E | 0.04641665 |
| BP | regulation of histone H3-K9 methylation | DNMT1/PAX5 | 0.04641665 |
| BP | mitochondrial depolarization | PARP1/GOT1 | 0.04641665 |
| BP | ATP biosynthetic process | FBP1/LDHC/LDHA/PARP1 | 0.048478871 |
| BP | skeletal muscle tissue development | PAX5/SOX17/FLNB/ACTA1 | 0.048478871 |
| BP | nucleoside monophosphate metabolic process | FBP1/UQCRC1/ADK/LDHC/LDHA/PARP1 | 0.049223655 |
| BP | regulation of actin filament organization | CORO1A/RAC1/ROCK2/HCK/NF2 | 0.049223655 |
| BP | negative regulation of JAK-STAT cascade | PPP2R1A/NF2 | 0.049253272 |
| CC | actin filament | CORO1A/RAC1/HCK/DAPK3/ACTA1 | 0.014508908 |
| CC | phagocytic vesicle | CORO1A/B2M/TLR1/FLNB/APPL1 | 0.016442798 |
| CC | mitochondrial matrix | GSTK1/IDH2/ACADS/PDHA1/GSTZ1/SCO2/PDK2/PCCA | 0.023244649 |
| CC | lamellipodium | CORO1A/RAC1/NF2/ACTA1/DPP4 | 0.023244649 |
| CC | focal adhesion | B2M/RAC1/HCK/FLNB/ANXA5/CHP1/DPP4 | 0.023244649 |
| CC | cell-substrate adherens junction | B2M/RAC1/HCK/FLNB/ANXA5/CHP1/DPP4 | 0.023244649 |
| CC | peroxisomal matrix | GSTK1/DAO/GRHPR | 0.023244649 |
| CC | microbody lumen | GSTK1/DAO/GRHPR | 0.023244649 |
| CC | cell-substrate junction | B2M/RAC1/HCK/FLNB/ANXA5/CHP1/DPP4 | 0.023244649 |
| CC | endocytic vesicle | CORO1A/B2M/TLR1/FLNB/APPL1/DPP4 | 0.023244649 |
| CC | peroxisome | GSTK1/IDH2/DAO/GRHPR | 0.031220764 |
| CC | microbody | GSTK1/IDH2/DAO/GRHPR | 0.031220764 |
| CC | contractile fiber | MMP2/SCO2/FLNB/CDK5R1/ACTA1 | 0.033796364 |
| CC | transcription factor complex | XRCC6/RARA/THRB/POU2F1/PARP1/SOX17 | 0.042427319 |
| CC | phagocytic vesicle membrane | CORO1A/B2M/TLR1 | 0.044051748 |
| MF | coenzyme binding | IDH2/KYNU/ACADS/LDHA/PARP1/DAO/GOT1/GRHPR/PCCA | 0.001215016 |
| MF | NAD binding | IDH2/LDHA/PARP1/GRHPR | 0.01402428 |
| MF | oxidoreductase activity, acting on CH-OH group of donors | IDH2/LDHC/LDHA/GRHPR/LIPF | 0.021524246 |
| MF | cadherin binding | ASAP1/NUDC/LDHA/PUF60/FLNB/CDK5R1/RAN | 0.039168133 |
| MF | protein kinase B binding | RARA/APPL1 | 0.043911217 |
| MF | nuclear receptor activity | RARA/THRB/AR | 0.043911217 |
| MF | transcription factor activity, direct ligand regulated sequence-specific DNA binding | RARA/THRB/AR | 0.043911217 |
| MF | cell adhesion molecule binding | ASAP1/NUDC/LDHA/PUF60/FLNB/VWF/CDK5R1/RAN | 0.043911217 |
| MF | carboxylic acid binding | RARA/CRABP2/GOT1/GRHPR/PCCA | 0.043911217 |
| MF | oxidoreductase activity, acting on the CH-OH group of donors, NAD or NADP as acceptor | IDH2/LDHC/LDHA/GRHPR | 0.043911217 |
| MF | steroid hormone receptor activity | RARA/THRB/AR | 0.043911217 |
| MF | organic acid binding | RARA/CRABP2/GOT1/GRHPR/PCCA | 0.043911217 |
| MF | transferase activity, transferring alkyl or aryl (other than methyl) groups | GSTK1/GSTZ1/SRM | 0.043911217 |
| MF | Ras GTPase binding | GDI1/VAV2/RAC1/ROCK2/DAPK3/RAPGEF4/RIMS2 | 0.047495744 |
| MF | monocarboxylic acid binding | RARA/CRABP2/PCCA | 0.047495744 |
| MF | small GTPase binding | GDI1/VAV2/RAC1/ROCK2/DAPK3/RAPGEF4/RIMS2 | 0.047495744 |
| MF | vitamin binding | KYNU/CRABP2/GOT1/PCCA | 0.047495744 |
| MF | retinoic acid binding | RARA/CRABP2 | 0.047495744 |
| MF | myosin binding | CORO1A/RAB6A/ACTA1 | 0.04971864 |
| MF | glutathione peroxidase activity | GSTK1/GSTZ1 | 0.04971864 |
| MF | triglyceride lipase activity | PNLIP/LIPF | 0.04971864 |

BP: biological process; CC: cellular component; MF: molecular function.

Table S2: KEGG pathways enrichment analysis of target genes of goserelin

| **ID** | **Description** | **Gene** | **P adjust** |
| --- | --- | --- | --- |
| hsa00270 | Cysteine and methionine metabolism | DNMT1/ENOPH1/LDHC/LDHA/GOT1/SRM | 0.000274191 |
| hsa01200 | Carbon metabolism | FBP1/IDH2/ACADS/PDHA1/GOT1/PCCA/ACO1 | 0.002267123 |
| hsa00640 | Propanoate metabolism | ACADS/LDHC/LDHA/PCCA | 0.006404882 |
| hsa00620 | Pyruvate metabolism | PDHA1/LDHC/LDHA/GRHPR | 0.008276912 |
| hsa01210 | 2-Oxocarboxylic acid metabolism | IDH2/GOT1/ACO1 | 0.010059189 |
| hsa00020 | Citrate cycle (TCA cycle) | IDH2/PDHA1/ACO1 | 0.029271809 |
| hsa00630 | Glyoxylate and dicarboxylate metabolism | GRHPR/PCCA/ACO1 | 0.029271809 |
| hsa04670 | Leukocyte transendothelial migration | MMP2/VAV2/RAC1/ROCK2/RAPGEF4 | 0.029271809 |
| hsa00010 | Glycolysis / Gluconeogenesis | FBP1/PDHA1/LDHC/LDHA | 0.029271809 |
| hsa05230 | Central carbon metabolism in cancer | PDHA1/LDHC/LDHA/SCO2 | 0.029271809 |
| hsa00350 | Tyrosine metabolism | HPD/GSTZ1/GOT1 | 0.036236667 |
